# Targeting Sirtuins in Neurodegenerative and Psychiatric Disorders: From Molecular Mechanisms to Pharmacological Modulation

**DOI:** 10.3390/neurolint18100189

**Published:** 2026-10-10

**Authors:** Andrei Florin Păduraru, Raluca-Sînziana Zaharia, Iuliana Roată, Lucian Hritcu, Razvan Stefan Boiangiu, Alin-Mihnea Lupașcu, Mădălina-Maria Popescu-Brezuleanu, Carmen-Olguța Brezuleanu

**Affiliations:** 1 Department for Teacher Training, Ion Ionescu de la Brad University of Life Sciences, 3 Mihail Sadoveanu Alley, 700490 Iasi, Romania; olguta.brezuleanu@iuls.ro; 2 Department of Pharmacognosy, Grigore T. Popa University of Medicine and Pharmacy, 16 Universitatii Street, 700115 Iasi, Romania; 3 Doctoral School of Engineering Sciences, Ion Ionescu de la Brad University of Life Sciences, 3 Mihail Sadoveanu Alley, 700490 Iasi, Romania; mihnea.lupascu@stud.iuls.ro; 4 Roxbury Elementary School, Stamford Public Schools, 751 West Hill Road, Stamford, CT 06902, USA; iroata@stamfordpublicschools.org; 5 Faculty of Biology, Alexandru Ioan Cuza University of Iasi, 700506 Iasi, Romania; hritcu@uaic.ro (L.H.); razvan.boiangiu@uaic.ro (R.S.B.); 6 Engineering and Management Department, Faculty of Industrial Design and Business Management, Gheorghe Asachi Technical University of Iasi, 71 A Mangeron Blvd, 700050 Iasi, Romania; madalina-maria.brezuleanu@academic.tuiasi.ro

**Keywords:** SIRT1, SIRT2, SIRT3, SIRT4, SIRT5, SIRT6, SIRT7, NAD^+^ metabolism, mitochondrial dysfunction, neuroinflammation

## Abstract

Neurological and psychiatric disorders share several biological mechanisms, including mitochondrial dysfunction, oxidative stress, neuroinflammation, impaired protein homeostasis, and changes in synaptic plasticity. Sirtuins (SIRT) are nicotinamide adenine dinucleotide (NAD^+^)-dependent enzymes that regulate cellular metabolism, gene expression, mitochondrial function, inflammation, and neuronal survival. This narrative review examines the roles of SIRT1–SIRT7 in neurodegenerative and psychiatric disorders and discusses pharmacological approaches that target sirtuins or related pathways. The review was complemented by a bibliometric analysis of publications indexed in the Web of Science Core Collection. Keyword co-occurrence analysis was used to identify the main themes and emerging research directions in the field. Current evidence suggests that activation of SIRT1 and SIRT3 generally supports mitochondrial function and neuronal resistance to stress, whereas inhibition of SIRT2 may be beneficial in some models of protein aggregation and cerebral ischemia. However, the effects of sirtuins vary according to the isoform, cell type, disease, and stage of disease. Pharmacological modulators were grouped into targeted activators, natural compounds that indirectly support sirtuin signaling, NAD^+^-boosting agents, synthetic inhibitors, and endogenous inhibitors with context-dependent effects. Among the more selective activators, SRT2104 and MDL-800 appear particularly relevant, while most natural compounds act as indirect pleiotropic modulators of sirtuin-associated signaling, although their translational validation remains limited. Overall, sirtuin modulation is a promising but complex therapeutic approach. Its clinical translation is still limited by poor isoform selectivity, indirect mechanisms of action, dose- and timing-dependent effects, uncertain blood–brain barrier penetration and insufficient clinical validation.

## 1. Introduction

Neurological and psychiatric disorders constitute a major and continuously expanding public-health burden. In 2021, more than three billion people worldwide were living with a neurological condition, while disorders of the nervous system became the leading global cause of health loss and disability. The total burden, expressed as disability-adjusted life years, increased by approximately 18% between 1990 and 2021 [1]. In parallel, mental disorders remain major contributors to disability worldwide, with depressive and anxiety disorders accounting for a substantial proportion of the global psychiatric burden [2]. Population ageing, longer life expectancy, population growth, and persistent inequalities in access to diagnosis and treatment are expected to further increase their clinical and socioeconomic impact [1].

Neurodegenerative and psychiatric disorders have traditionally been investigated as distinct diagnostic categories. Nevertheless, accumulating evidence indicates that they share several interconnected biological abnormalities, including mitochondrial dysfunction, oxidative stress, impaired proteostasis, chronic neuroinflammation, altered synaptic plasticity, disrupted energy metabolism, and epigenetic dysregulation [3,4]. These convergent mechanisms are relevant because cognitive decline, affective symptoms, anxiety, sleep disturbances, and behavioural alterations frequently coexist in patients with neurological disease [5]. Conversely, metabolic, inflammatory, and neuroplasticity-related abnormalities are increasingly recognized in psychiatric disorders [6]. An integrated neuropsychiatric perspective may therefore provide a more biologically informative framework than approaches limited to conventional diagnostic boundaries.

Sirtuins are a highly conserved family of NAD^+^-dependent enzymes that connect cellular metabolic status with post-translational protein regulation. Seven mammalian isoforms, SIRT1-SIRT7, have been identified. Although they possess a conserved catalytic domain, these enzymes differ substantially in their subcellular localization, enzymatic activity, substrate specificity, and physiological functions. SIRT1, SIRT6, and SIRT7 are predominantly nuclear, although SIRT1 can shuttle between the nucleus and cytoplasm; SIRT2 is primarily cytoplasmic but may translocate to the nucleus; and SIRT3, SIRT4, and SIRT5 are mainly mitochondrial [7,8]. This spatial and functional diversity enables sirtuins to regulate multiple cellular compartments and biological processes.

Through isoform-specific lysine deacylation reactions, including deacetylation, desuccinylation, demalonylation, deglutarylation, and long-chain fatty-acyl removal, sirtuins regulate histone and non-histone substrates involved in transcription, mitochondrial metabolism, DNA (deoxyribonucleic acid) repair, oxidative-stress responses, autophagy, inflammation, apoptosis, and cellular survival. Their enzymatic dependence on NAD^+^ gives them an important role as metabolic sensors capable of translating changes in nutrient availability, cellular energy, and redox state into adaptive molecular responses. Since NAD^+^ availability decreases or becomes dysregulated during ageing, inflammation, oxidative injury, and mitochondrial dysfunction, alterations in NAD^+^-sirtuin signalling may contribute directly to disease progression [9].

The relevance of this enzymatic family to brain disorders is reinforced by the high energetic demands and limited regenerative capacity of neural tissue. Neurons depend strongly on mitochondrial adenosine triphosphate (ATP) production, redox stability, efficient axonal transport, protein quality control, and precisely regulated synaptic plasticity [10,11]. In glial cells and astrocytes, sirtuins influence metabolic homeostasis, oxidative-stress responses, and inflammatory signalling [12]. Sirtuins are positioned at the intersection of these processes and can influence neuronal survival, glial activation, synaptic function, memory formation, stress adaptation, and behavioural regulation. Consequently, impaired or maladaptive sirtuin signalling may participate in both neurodegenerative pathology and psychiatric vulnerability [9].

The therapeutic relevance of sirtuins is highly context-dependent and cannot be reduced to a uniform strategy of activation or inhibition. In selected experimental models, increased SIRT1 or SIRT3 signaling has been associated with improved mitochondrial function, antioxidant defense, and neuronal resilience, whereas SIRT2 inhibition has reduced neuronal injury in some proteotoxic and ischemic settings. However, these effects vary according to brain region, neuronal or glial cell type, disease stage, metabolic state, subcellular localization, treatment timing and duration, and, in some models, sex. For example, SIRT2 inhibition may reduce neuronal toxicity while simultaneously interfering with protective functions in microglia, oligodendrocytes, or peripheral immune cells. Accordingly, the direction and therapeutic value of sirtuin modulation should be interpreted within the specific disease and cellular context rather than generalized across neurological and psychiatric disorders [7,9].

Despite extensive preclinical research, the clinical translation of sirtuin-targeted interventions remains limited. Many compounds described as sirtuin modulators are pleiotropic molecules that also affect inflammatory pathways, oxidative stress, mitochondrial function, and autophagy. Consequently, it is often difficult to determine whether an observed neuroprotective effect results from direct modulation of a specific sirtuin or from broader metabolic and antioxidant actions. Additional limitations include low bioavailability, uncertain blood–brain barrier penetration, incomplete pharmacokinetic characterization, dose-dependent toxicity, limited evidence that the compounds effectively reach and modulate their intended targets, and a lack of validated biomarkers for assessing sirtuin activity in the human brain [13].

## 2. Scope and Objective of the Review

This review aims to provide an integrated analysis of the molecular and pharmacological relevance of the mammalian sirtuin family, SIRT1-SIRT7, in neurodegenerative and psychiatric disorders.

Firstly, a complementary bibliometric analysis is incorporated to characterize the development and conceptual organization of research on sirtuins and their pharmacological modulation in neurological and neuropsychiatric contexts. Publication trends, disciplinary distribution, keyword co-occurrence, thematic clusters, and temporal changes are examined to identify established research domains and emerging directions. This analysis is intended to support the narrative synthesis by illustrating the transition from fundamental studies of NAD^+^-dependent deacetylation and cellular metabolism toward disease-oriented research, isoform-selective modulation, molecular docking and the development of therapeutic sirtuin modulators.

Secondly, the review examines the biological functions of SIRT1-SIRT7 in the central nervous system and evaluates the experimental evidence linking individual sirtuin isoforms to neurodegenerative disorders, including Alzheimer’s disease, Parkinson’s disease, Huntington’s disease, cerebral ischemia, and other forms of neurological injury.

The third objective is to critically evaluate natural and synthetic compounds that modulate sirtuin-associated pathways. These include direct activators and inhibitors, NAD^+^ precursors, and pleiotropic natural compounds that affect sirtuin activity indirectly through AMP-activated protein kinase (AMPK) signaling, NAD^+^ metabolism, mitochondrial regulation, antioxidant pathways, autophagy, or anti-inflammatory mechanisms. In the end, the review seeks to identify the principal translational limitations of current sirtuin-targeted strategies.

By integrating experimental disease models, selected clinical data, and bibliometric trends, this review aims to clarify the therapeutic potential and current limitations of sirtuin modulation in neuropsychiatric diseases.

## 3. Literature Search and Bibliometric Overview

A bibliometric analysis was conducted as a complementary component of the narrative review to characterize the conceptual structure, thematic organization, and temporal evolution of research on sirtuins and their pharmacological modulation in neuropsychiatric disorders. Bibliographic records were retrieved from the Web of Science Core Collection. The search strategy was designed to capture publications addressing the SIRT1-SIRT7 family, sirtuin-related molecular mechanisms, neurodegenerative and psychiatric disorders, and pharmacological sirtuin modulators.

The following search query was applied to the topic field:

(sirtuin* OR SIRT1 OR SIRT2 OR SIRT3 OR SIRT4 OR SIRT5 OR SIRT6 OR SIRT7) AND (brain OR neural OR neuron* OR neurodegenerat* OR neuropsychiatr* OR psychiatr* OR cognit* OR depress* OR anxiety OR mood OR Alzheimer OR Parkinson).

The asterisk (*) was used as a truncation operator to retrieve multiple lexical variants sharing the same word stem, thereby increasing the sensitivity and comprehensiveness of the search strategy.

The search was conducted in June 2026 and covered publications indexed between 2003 and 2026. For the longitudinal analysis, only publications from 2003 to 2025 were included, as 2003 was the earliest publication year represented in the retrieved dataset. Publications from 2026 were excluded because the year was incomplete at the time of data retrieval.

The results were refined according to document type (article or review).

To obtain a pharmacologically focused subset, the search strategy was further refined by adding the following topic-field block:

AND (modulat* OR activat* OR inhibit* OR agonist* OR antagonist* OR pharmacolog* OR therapeut* OR molecule* OR drug discovery OR molecular docking).

After applying this additional search refinement, 3888 publications were retained for the pharmacological bibliometric analysis.

Descriptive analyses, including annual publication trends and Web of Science category distributions, were performed using the complete pharmacologically refined dataset of 3888 publications.

The records were ranked according to the Web of Science relevance criterion, and the first 1000 records were exported as full records and cited references for bibliometric analysis using VOSviewer (version 1.6.20). Keyword co-occurrence analysis was performed to identify the principal conceptual relationships, thematic structures, and emerging research directions within the pharmacologically focused literature.

Keywords associated with the sirtuin family, molecular and epigenetic mechanisms, NAD^+^ metabolism, neuroprotection, inflammation, depression, anxiety, neurodegeneration, and pharmacological modulation were included in the analysis. Of the 3670 keywords identified in the selected dataset, 372 met this criterion and were retained for bibliometric mapping.

Prior to network construction, terminology was standardized to reduce fragmentation caused by spelling variants, singular and plural forms, abbreviations, and alternative nomenclature. Equivalent terms, including “sirtuin” and “sirtuins”, “SIRT-1” and “SIRT1”, and “Alzheimer disease” and “Alzheimer’s disease”, were merged where appropriate. Non-specific or methodologically uninformative terms were excluded when they did not contribute to the conceptual interpretation of the field.

Two visualization modes were used. Network visualization was applied to identify thematic clusters and relationships among the most prominent keywords. Overlay visualization was used to examine the temporal evolution of research topics based on the average publication year associated with each keyword.

In the generated maps, node size represents keyword occurrence frequency, whereas links indicate co-occurrence relationships between terms. Link thickness reflects the strength of association, and spatial proximity indicates greater conceptual relatedness. Given the high density of the resulting network, the interpretation focused on terms directly relevant to the scope of the review, including sirtuins, individual SIRT isoforms, deacetylation, oxidative stress, neuroinflammation, neurodegenerative disorders, depression, anxiety, and pharmacological modulators.

### 3.1. Web of Science Database Analysis

**Figure 1:**
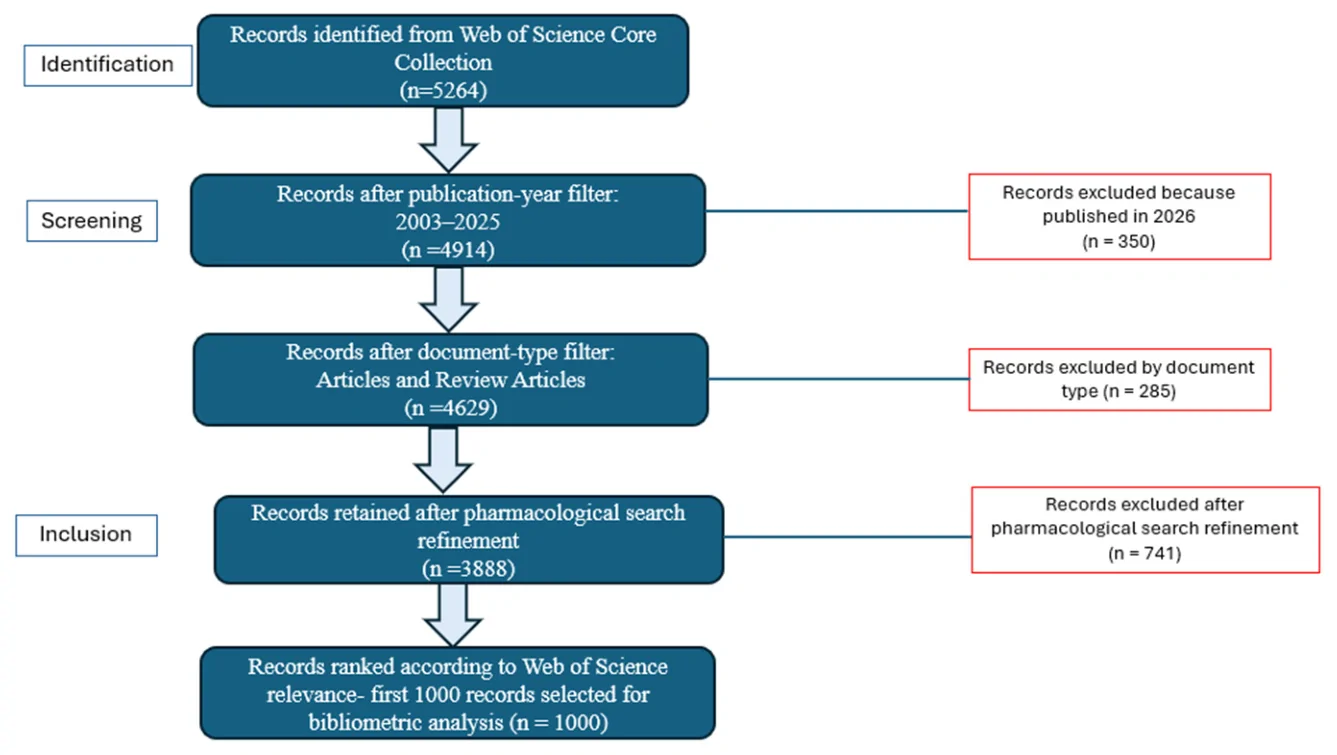
Flow diagram of bibliometric data retrieval and selection.

Figure 1 summarizes the bibliometric data retrieval and selection process. A total of 5264 records were initially identified in the Web of Science Core Collection. After restricting the publication period to 2003–2025, 4914 records were retained, while 350 publications from 2026 were excluded because the year was incomplete at the time of data retrieval. The dataset was subsequently restricted to articles and review articles, resulting in 4629 eligible records; 285 publications belonging to other document types were excluded.

To obtain a pharmacologically focused dataset, the initial search strategy was further refined using terms related to sirtuin modulation, activation, inhibition, pharmacology, therapeutics, small molecules, drug discovery, and molecular docking. This refinement yielded 3888 publications, while 741 records were excluded because they did not meet the pharmacological search criteria. The retained records were then ranked according to the Web of Science relevance criterion, and the first 1000 publications were selected and exported as full records and cited references for keyword co-occurrence analysis in VOSviewer.

**Figure 2:**
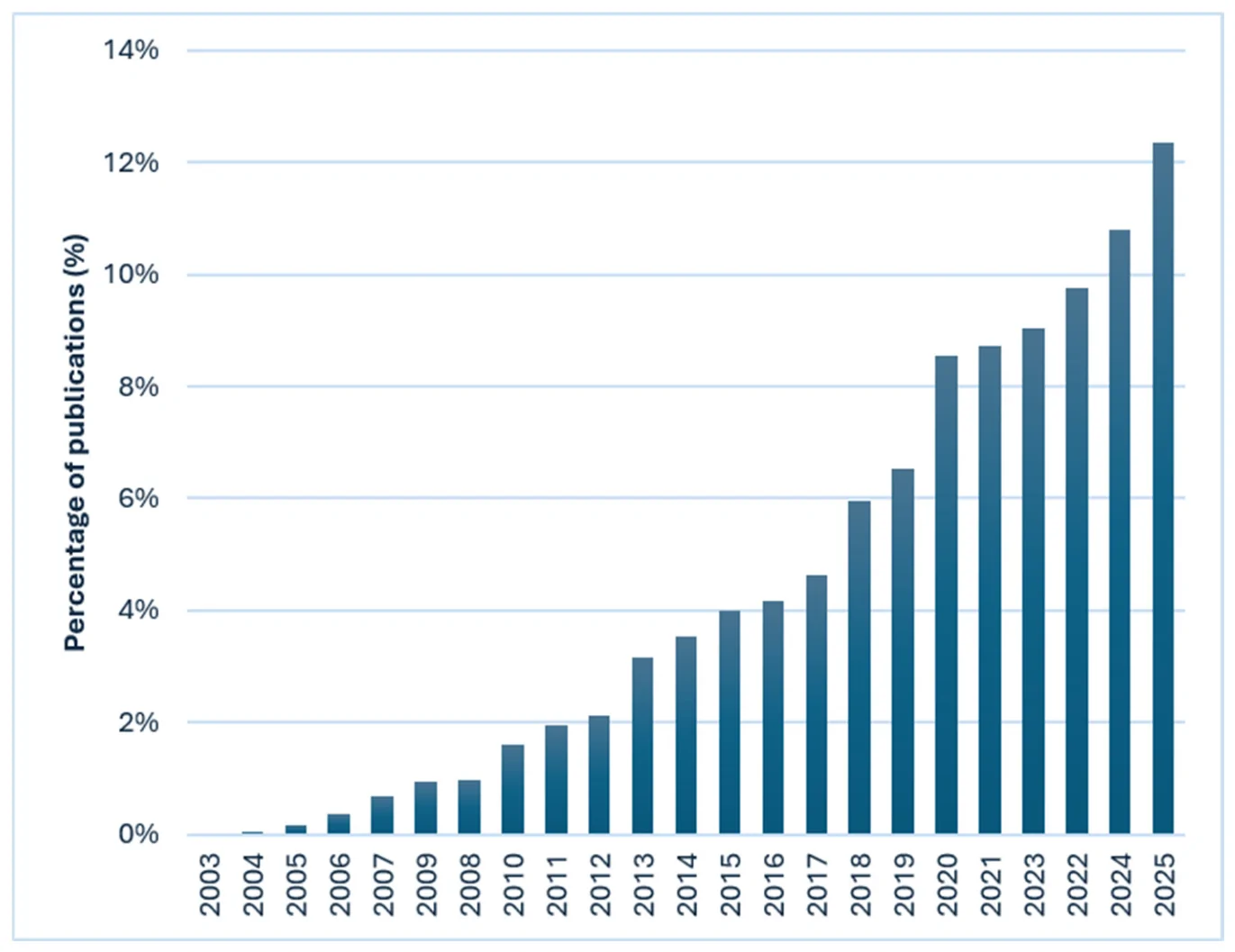
Annual percentage distribution of publications included in the pharmacologically refined bibliometric dataset on sirtuins in neurological and neuropsychiatric research, indexed in the Web of Science Core Collection from 2003 to 2025.

The annual distribution of the 3888 publications included in the pharmacologically refined dataset showed a marked and sustained increase in scientific output between 2003 and 2025. Publications represented only a very small proportion of the dataset during the initial years, remaining below 1% annually until approximately 2009. A gradual increase became evident after 2010, followed by a more pronounced acceleration from 2017 onward (Figure 2).

The highest annual proportions were recorded in the most recent complete years, with publication output exceeding 8% of the total dataset from 2020 onward and reaching its maximum in 2025, when publications accounted for approximately 12% of all records included in the analysis. This pattern indicates a substantial expansion of research interest in the pharmacological modulation of sirtuins in neurological and neuropsychiatric contexts, particularly during the last decade. Publications from 2026 were excluded from the longitudinal analysis because the search was conducted in June 2026 and the publication year was therefore incomplete.

**Figure 3:**
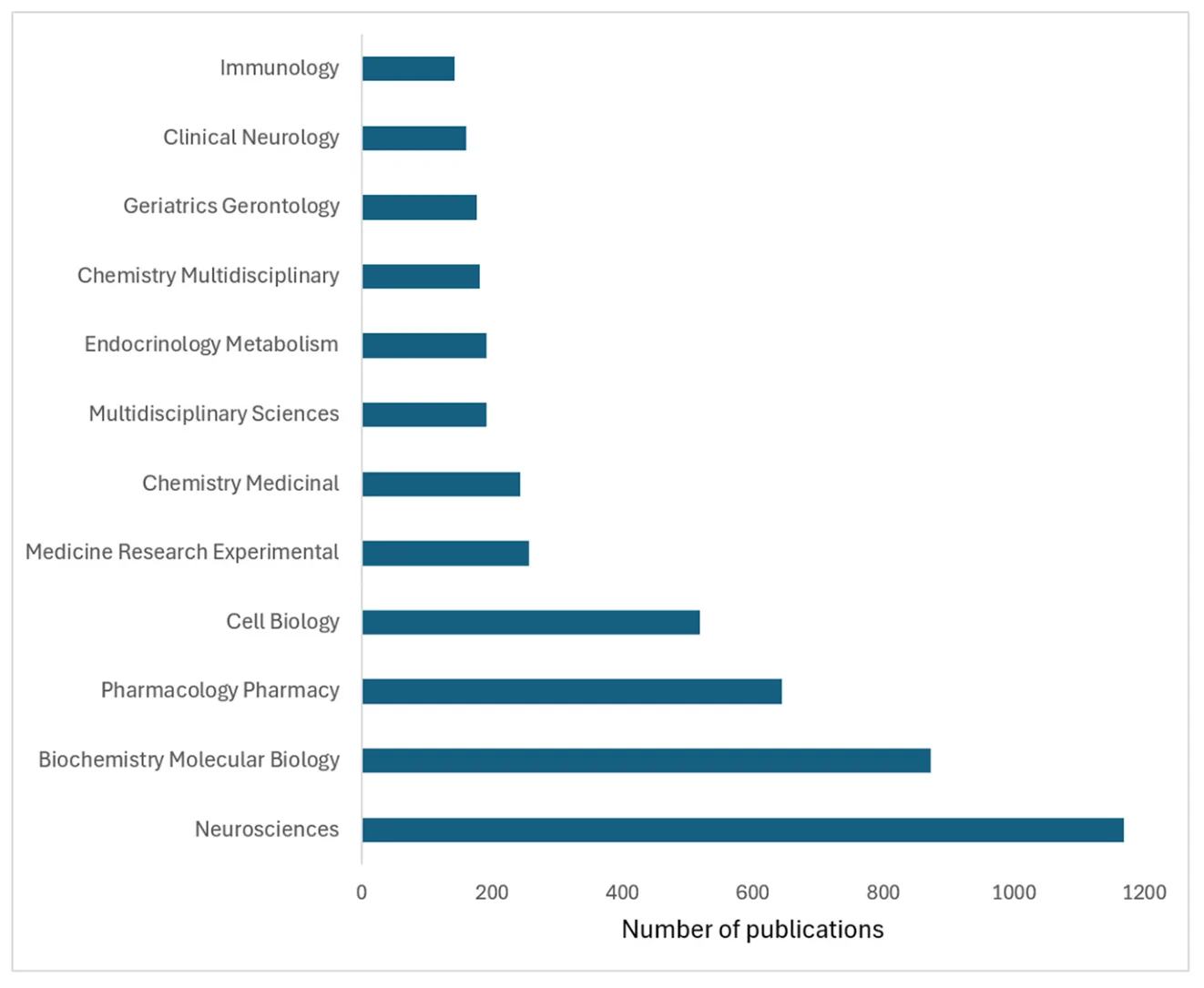
Distribution of publications included in the pharmacologically refined bibliometric dataset across the leading Web of Science categories, 2003–2025.

The distribution of the 3888 publications included in the pharmacologically refined dataset across Web of Science categories is presented in Figure 3. For improved readability, the graphical representation was limited to the top 12 Web of Science categories ranked by number of publications. The remaining categories were not displayed because each comprised fewer than 100 publications. Neurosciences was the predominant category, followed by Biochemistry and Molecular Biology, Pharmacology and Pharmacy, Cell Biology, and Medicine, Research and Experimental. This distribution highlights the multidisciplinary nature of research on the pharmacological modulation of sirtuins in neurological and neuropsychiatric contexts, integrating neuroscience, molecular biology, pharmacology, experimental medicine, medicinal chemistry, metabolism, and ageing research. Because Web of Science categories are assigned at the journal level, individual publications may be assigned to more than one category; therefore, category counts are not mutually exclusive.

### 3.2. Bibliometric Mapping Using VosViewer

**Figure 4:**
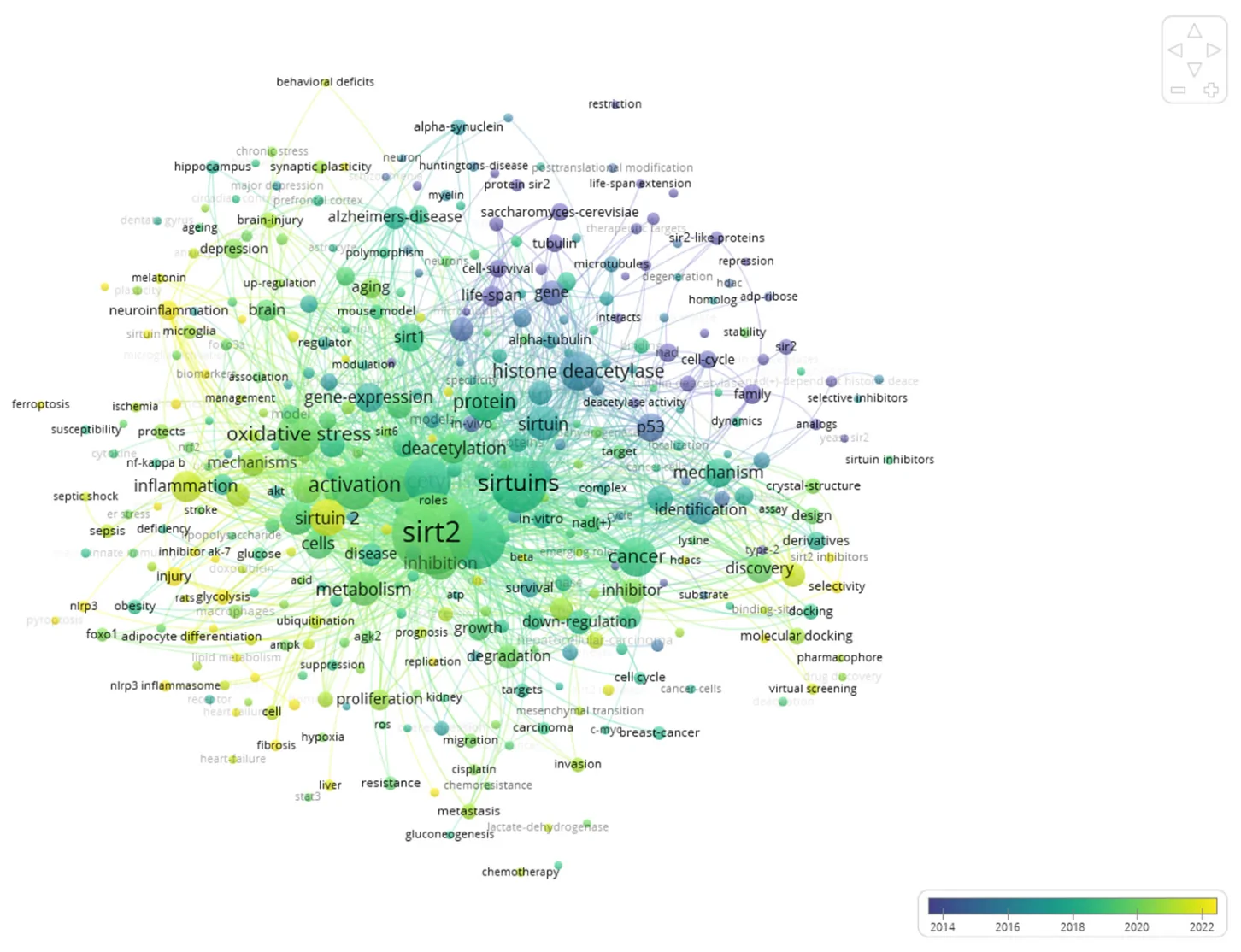
Temporal overlay visualization of keyword co-occurrence in research on sirtuins and their pharmacological modulation. Node colors represent the average publication year.

The temporal overlay of the keyword co-occurrence network related to sirtuins and their pharmacological modulation is illustrated in Figure 4. The visualization highlights the main conceptual connections among molecular mechanisms, neurological and neuropsychiatric disorders, and pharmacological research directions. Larger nodes represent more frequently occurring keywords, whereas the links and their spatial arrangement reflect the strength of co-occurrence and conceptual proximity. The color scale indicates the average publication year, with blue representing earlier topics and yellow indicating more recent areas of interest.

The overlay visualization revealed a gradual evolution of the research field from fundamental molecular studies toward disease-oriented and pharmacological investigations. Earlier research topics were associated with experimental models such as *Saccharomyces cerevisiae* and with concepts including histone deacetylation, α-tubulin, microtubules, transcriptional regulation, and cellular metabolism. These terms reflect the initial characterization of sirtuins as NAD^+^-dependent enzymes and regulators of protein acetylation.

Intermediate research topics increasingly addressed oxidative stress, metabolism, SIRT2 activity, Alzheimer’s disease, brain injury, and depression, indicating the expansion of sirtuin research toward neurological and neuropsychiatric disorders. More recent terms, including “sirtuin inhibitors”, “molecular docking”, “discovery”, “derivatives”, “crystal structure”, and “selectivity”, suggest a transition toward pharmacological modulation, structure-based compound design, and the development of more selective sirtuin-targeting agents. However, the presence of oncology-related terms also indicates that the analyzed literature remains multidisciplinary rather than exclusively focused on neuropsychiatric research.

**Figure 5:**
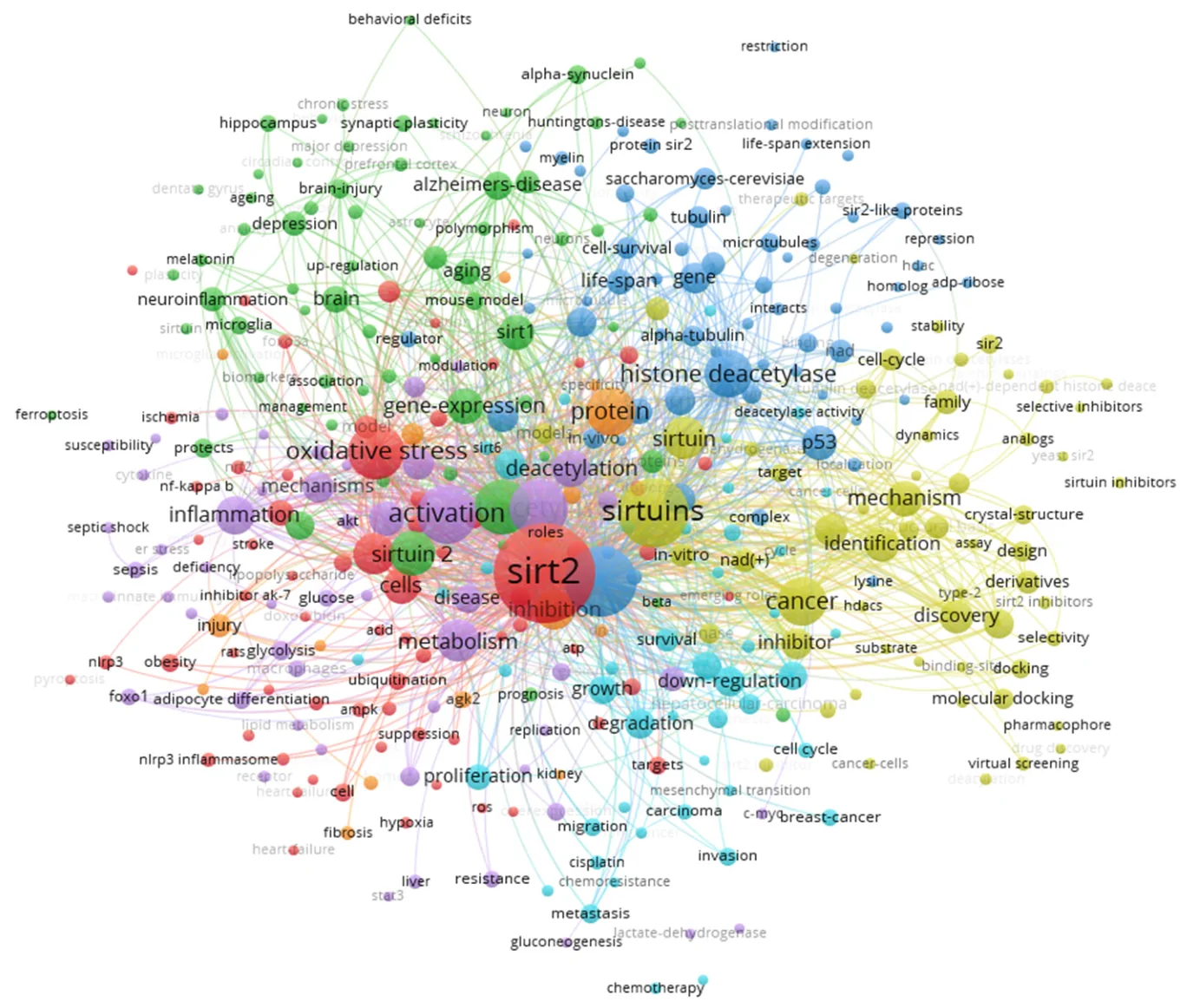
Network visualization of keyword co-occurrence in research on sirtuins and their pharmacological modulation. Node size indicates keyword frequency, links represent co-occurrence relationships, and colors indicate thematic clusters automatically generated by VOSviewer.

The keyword co-occurrence map (Figure 5) reveals a highly interconnected conceptual structure within the analyzed field. The most prominent and centrally positioned terms include sirtuins, SIRT2, sirtuin, deacetylation, activation, protein, histone deacetylase, oxidative stress, metabolism, autophagy, gene expression, inflammation, Alzheimer’s disease, brain injury, depression, and molecular docking.

A particularly notable feature is the central position of SIRT2, which appears as one of the largest and most highly connected nodes in the network. This suggests substantial research interest in this isoform and its involvement in multiple biological and pathological processes. At the same time, the presence of terms such as Alzheimer’s disease, brain injury, depression, neuroprotection, and neuroinflammation indicates that research on sirtuins has expanded beyond fundamental molecular biology toward neurological and neuropsychiatric applications.

The close association of Alzheimer’s disease with concepts such as oxidative stress, protein, gene expression, deacetylation, and neuroinflammation suggests that the literature increasingly addresses the role of sirtuins in protein aggregation, neuronal inflammation, mitochondrial dysfunction, and cognitive decline. This pattern is particularly relevant to the scope of the present review because it links sirtuin biology with mechanisms involved in cognition and neurodegeneration.

The term depression is less central than several neurodegeneration-related keywords, but its presence within the network points to a growing extension of sirtuin research toward affective disorders and mood regulation. This finding supports the inclusion of psychiatric outcomes in the current review and suggests that the relationship between sirtuin signaling, stress-related mechanisms, and emotional regulation is emerging as a distinct research direction.

The map also highlights an important pharmacological dimension through the presence of terms such as inhibitor, sirtuin inhibitors, molecular docking, derivatives, selectivity, and mechanism. These associations indicate that recent studies are no longer limited to the biological roles of sirtuins but increasingly focus on computational screening, rational compound design, and the development of more selective modulators of individual SIRT isoforms.

**Figure 6:**
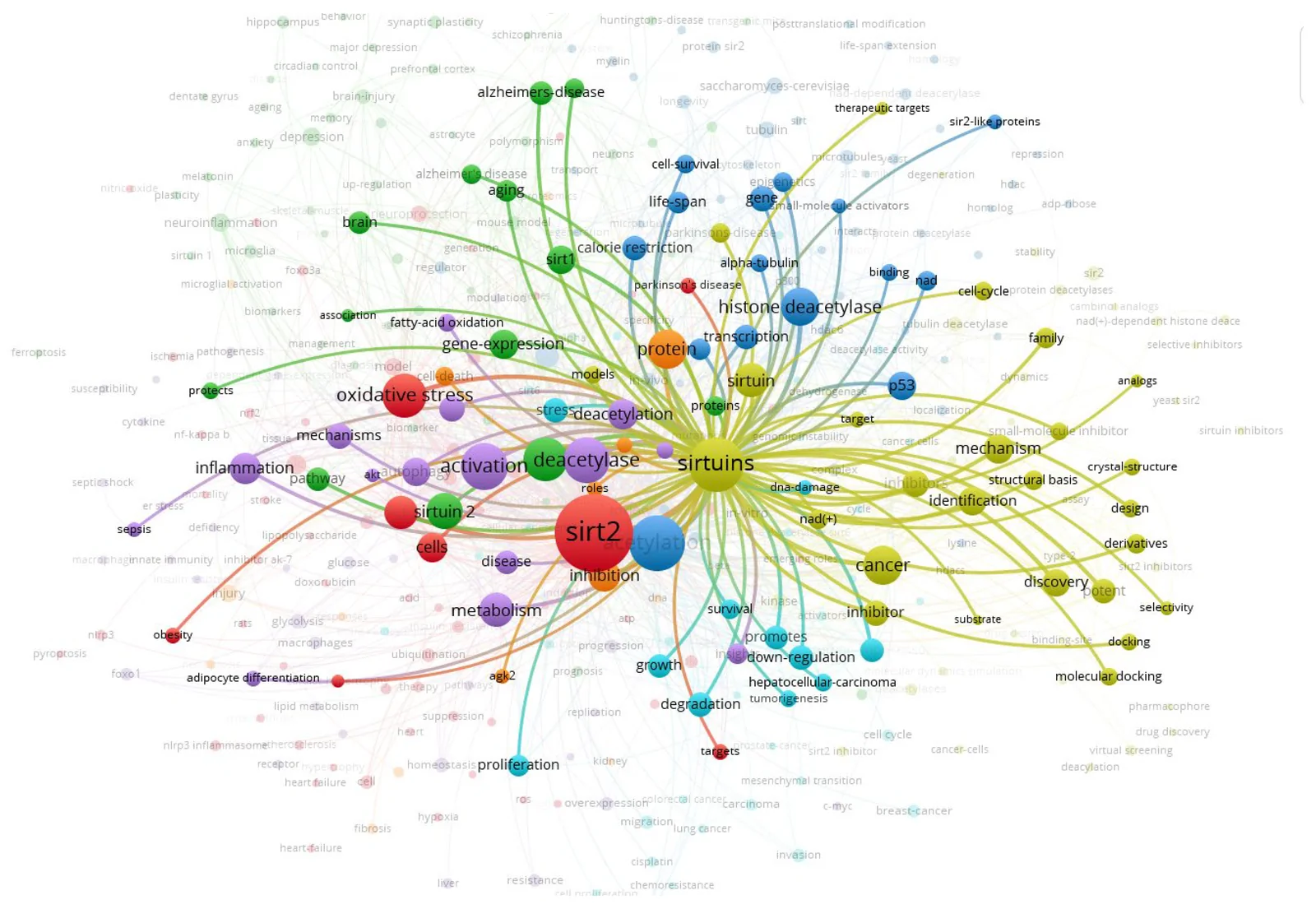
First cluster—sirtuins, pharmacological modulators, and molecular design. Node size indicates keyword frequency, links represent co-occurrence relationships, and colors indicate thematic substructures identified by VOSviewer.

Figure 6 presents a focused network visualization of the principal keyword relationships identified in the bibliometric dataset. Compared with the complete co-occurrence map, this representation emphasizes the most central and strongly connected terms, thereby facilitating the interpretation of the dominant thematic directions and the relationships between molecular mechanisms, disease-related concepts, and pharmacological research.

The first cluster includes terms such as sirtuins, sirtuin, mechanism, inhibitors, sirtuin inhibitors, discovery, molecular docking, design, derivatives, and crystal structure. Together, these keywords define a clear pharmacological and medicinal-chemistry-oriented research direction.

This cluster indicates that sirtuin research is no longer limited to the characterization of biological mechanisms, but increasingly focuses on the identification and rational design of molecules capable of modulating sirtuin activity. The close association between terms such as molecular docking, crystal structure, derivatives, and design suggests a growing emphasis on structure-based drug discovery, virtual screening, compound optimization, and the development of more selective modulators of individual SIRT isoforms.

This cluster is particularly relevant to the present review because it directly supports the section devoted to sirtuin modulators. However, the predominance of terms related to molecular design, docking, discovery, and inhibitor development suggests that this research direction remains largely preclinical. Clinical translation, target engagement, pharmacokinetic characterization, and validated biomarkers appear to be less prominently represented within this thematic area.

**Figure 7:**
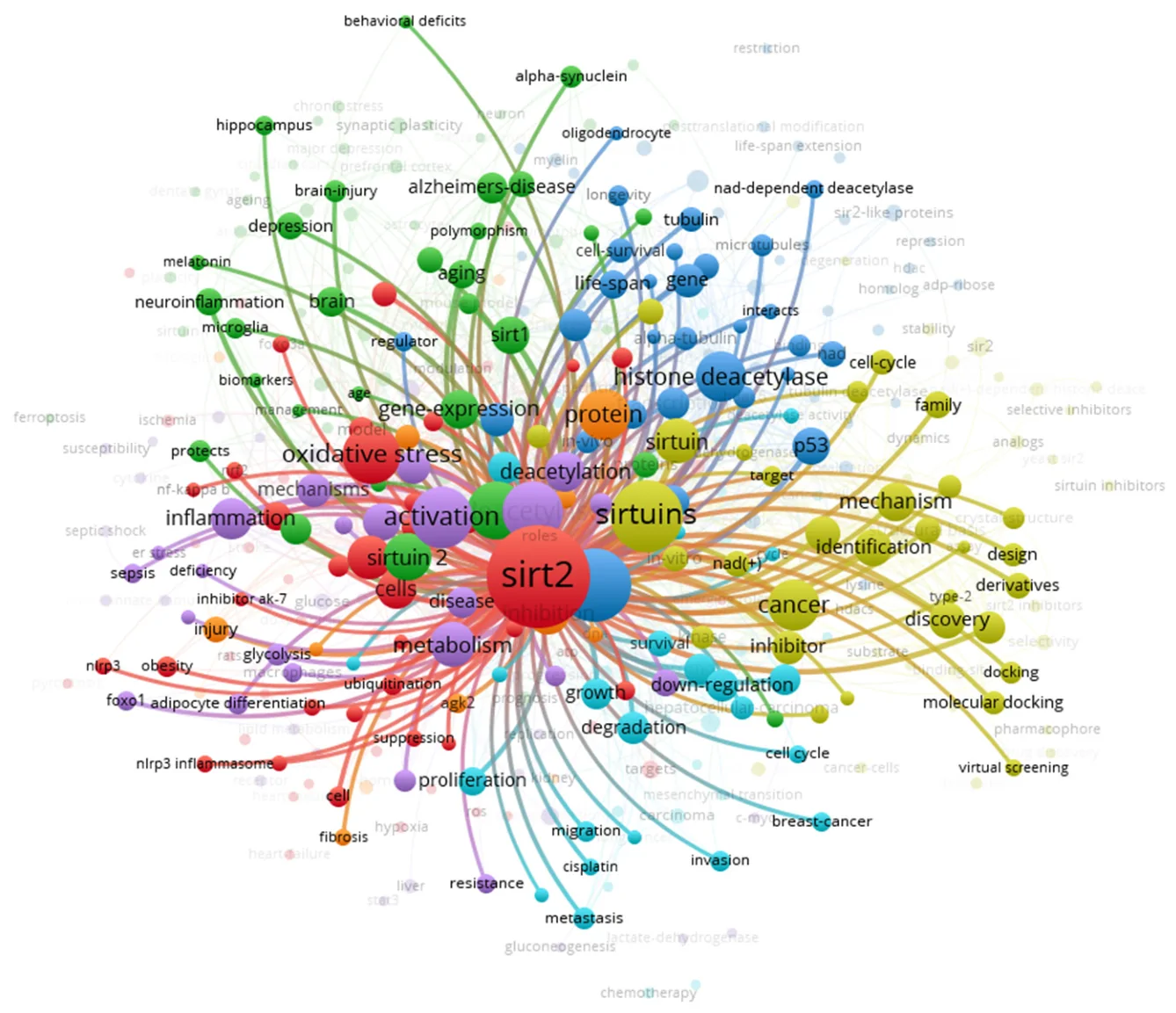
Second cluster—SIRT2, oxidative stress, inflammation, and cell death. Node size reflects keyword frequency, nodes sharing the same color belong to the same thematic substructures identified by VOSviewer, while spatial proximity reflects the strength of association between keywords.

One of the most prominent and centrally positioned thematic clusters is organized around SIRT2, which appears as one of the largest and most highly connected nodes in the network. SIRT2 is closely associated with terms such as oxidative stress, inflammation, metabolism, disease, apoptosis, inhibition, NLRP3 inflammasome, NF-κB, autophagy, ischemia, and pathogenesis (Figure 7).

The central position of SIRT2 indicates that this isoform is frequently investigated in relation to cellular stress responses, inflammatory signaling, metabolic dysfunction, programmed cell death, and pharmacological inhibition. Its strong connectivity suggests that SIRT2 functions as an important conceptual link between molecular mechanisms and disease-related processes across the analyzed literature.

The close relationship between oxidative stress and inflammation is particularly relevant to disorders of the central nervous system, because these mechanisms contribute to neuronal injury, mitochondrial dysfunction and altered cellular survival. Their association with apoptosis, autophagy, NLRP3 inflammasome, and NF-κB further highlights the role of SIRT2 in pathways involved in neurodegeneration, ischemic injury, and neuroinflammatory responses.

This cluster is also relevant to affective and stress-related disorders, in which persistent oxidative stress and dysregulated inflammatory signaling may influence neuronal plasticity, neurotransmission, and behavioral vulnerability. Overall, the cluster supports the interpretation of SIRT2 as a context-dependent regulator positioned at the intersection of metabolism, inflammation, cellular stress, and neuronal survival.

**Figure 8:**
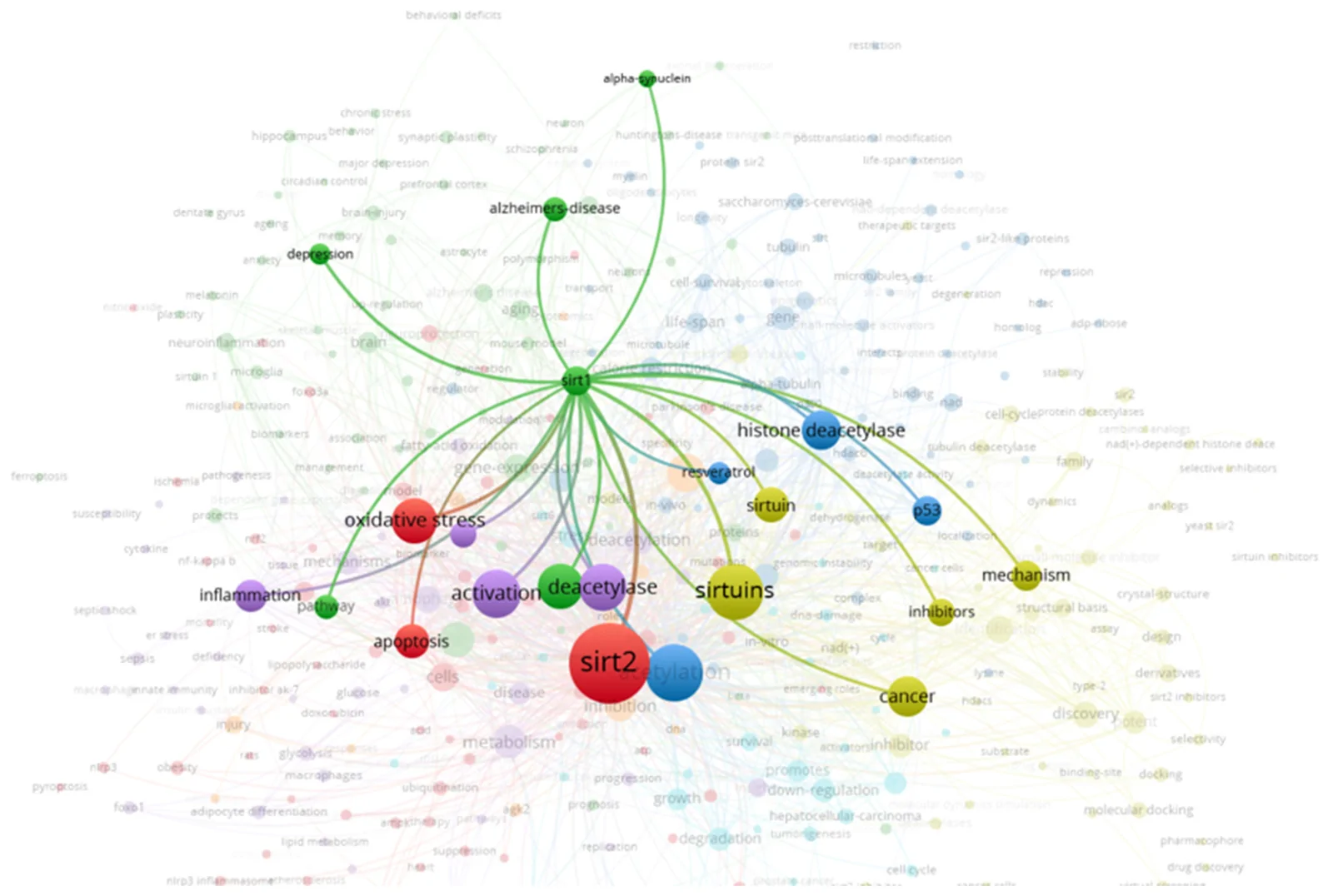
Third cluster—SIRT1, neurodegeneration, cognition, and mood regulation. Node size reflects keyword frequency, colors indicate indicate thematic substructures identified by VOSviewer, and the proximity between nodes reflect the strength of keyword associations.

Another important thematic cluster relevant to the scope of this review is centered on SIRT1, which is closely associated with keywords such as Alzheimer’s disease, depression, brain, aging, memory, hippocampus, synaptic plasticity, brain injury, neuroinflammation, anxiety, alpha-synuclein, and Parkinson’s disease (Figure 8).

This cluster is particularly relevant because it links cognition to mood regulation. The presence of terms such as Alzheimer’s disease, hippocampus, memory, and synaptic plasticity indicates that SIRT1 is frequently investigated in relation to cognitive function, brain aging, and neuronal plasticity.

At the same time, the occurrence of depression, anxiety, circadian control, and prefrontal cortex points to an expansion of sirtuin research into the field of biological psychiatry. These associations suggest that SIRT1-related mechanisms are being increasingly examined not only in neurodegeneration, but also in mood regulation, stress responses, circadian biology, and affective vulnerability.

Overall, this cluster positions SIRT1 at the intersection of neuroprotection, cognitive function, synaptic adaptation, neuroinflammation, and emotional regulation, thereby reinforcing the integrated neuropsychiatric perspective of the present review.

Taken together, the analyzed clusters indicate that sirtuins occupy a central position at the intersection of oxidative stress, cellular metabolism, epigenetic regulation, neuronal survival, and mood regulation. In the context of neurodegeneration, the generated maps reveal clear conceptual links among sirtuin-related research, Alzheimer’s disease, aging, oxidative stress, and neuroinflammatory mechanisms.

The bibliometric findings support the view that sirtuins are not only deacetylating enzymes but also regulators of several interconnected biological processes. They contribute to metabolic sensing, cellular stress responses, epigenetic regulation, mitochondrial function, neuronal plasticity, and inflammatory signaling. Through these roles, sirtuins connect basic molecular mechanisms with the development and progression of neurological and psychiatric disorders.

The bibliometric patterns identified above were used as a contextual framework for the narrative synthesis. Specifically, the prominence and co-occurrence of terms related to oxidative stress, inflammation, metabolism, SIRT2, Alzheimer’s disease, depression, molecular docking, and selectivity guided the organization of the subsequent discussion of isoform-specific mechanisms (Section 4) and pharmacological modulators (Section 5). The following sections therefore critically examine the experimental and clinical evidence underlying the thematic relationships highlighted by the bibliometric maps.

## 4. Sirtuin Isoforms and Their Roles in Neurodegenerative and Psychiatric Disorders

Although all mammalian sirtuins belong to the NAD^+^-dependent lysine deacylase family, their catalytic preferences differ substantially among isoforms. SIRT1, SIRT2, and SIRT3 function predominantly as deacetylases, whereas SIRT5 preferentially catalyzes desuccinylation, demalonylation, and deglutarylation. SIRT4 exhibits relatively weak classical deacetylase activity but possesses adenosine diphosphate (ADP)-ribosyltransferase, lipoamidase/delipoylase, and other lysine deacylase activities, while SIRT6 combines deacetylase activity with long-chain fatty-acyl lysine deacylation and mono-ADP-ribosyltransferase activity. SIRT7 exhibits deacetylase and desuccinylase activities, with additional deacylase functions reported [14]. Unlike zinc-dependent histone deacetylases, sirtuins require NAD^+^ for their catalytic activity, which links protein modification to the cellular energy and redox state. During deacetylation, they remove acyl groups from lysine residues and convert NAD^+^ into nicotinamide and O-acyl-ADP-ribose. Through this process, sirtuins regulate both histone and non-histone proteins involved in gene expression, metabolism, mitochondrial function, DNA repair, inflammation, oxidative stress, apoptosis, and cell survival [7,8,15].

Seven mammalian isoforms, SIRT1-SIRT7, have been identified. They share a conserved catalytic domain but differ in subcellular localization, enzymatic activity, substrate specificity, and biological function. SIRT1, SIRT6, and SIRT7 are predominantly nuclear, although SIRT1 can shuttle between the nucleus and cytoplasm. SIRT2 is primarily cytoplasmic but can translocate to the nucleus, particularly during mitosis or cellular stress. SIRT3, SIRT4, and SIRT5 are mainly mitochondrial and regulate metabolic and redox processes within this organelle. These distributions are not absolute and may vary according to cell type, developmental stage, and stress conditions [7,16].

SIRT1 is the most extensively studied isoform in the nervous system. It deacetylates transcription factors and regulatory proteins such as PGC-1α, FOXO proteins, p53, NF-κB, and components of the autophagic mechanism. Through these substrates, SIRT1 can support mitochondrial biogenesis, antioxidant defense, autophagy, DNA repair, and neuronal survival while limiting inflammatory and apoptotic signaling. SIRT1 also contributes to learning, memory, and synaptic plasticity. Genetic SIRT1 deficiency in mice impairs long-term potentiation and memory formation, whereas another study demonstrated that SIRT1 regulates synaptic plasticity. In addition, the lack of Sirt1 results in synaptic plasticity damage [17,18].

In preclinical Alzheimer’s disease models, early or preventive SIRT1 activation reduced amyloidogenic processing, promoted non-amyloidogenic cleavage of amyloid precursor protein (APP), and limited subsequent amyloid accumulation. In Tg2576 transgenic mice, a murine model of Alzheimer-like amyloid pathology, calorie restriction increased brain NAD^+^ and SIRT1 signaling and was associated with reduced Aβ accumulation. Moreover, increased SIRT1 activity reduced ROCK1 expression and enhanced α-secretase-mediated non-amyloidogenic processing of APP, thereby decreasing Aβ generation [19]. These effects may be more beneficial before major cellular damage occurs, when NAD^+^ metabolism and cellular function are still relatively preserved. In postmortem human frontal cortex from individuals with amnestic mild cognitive impairment, miR-212/132 and miR-23a/23b, which act as post-transcriptional negative regulators of SIRT1, were selectively reduced in the frontal cortex and SIRT1 expression was increased, suggesting a possible compensatory neuroprotective response during early disease stages. Further experiments in human neuronotypic cells showed that increased SIRT1 expression reduced Aβ1-42-induced toxicity, whereas pharmacological SIRT1 inhibition with EX-527 abolished this protection [20]. Together, these findings support a stage-dependent model in which SIRT1 may contribute to endogenous neuroprotective responses during early Alzheimer’s disease, while the therapeutic benefit of pharmacological SIRT1 activation in humans remains to be established.

SIRT2 is highly expressed in the brain, particularly in oligodendrocytes, but is also present in neurons and glial cells. It deacetylates cytosolic proteins such as α-tubulin, thereby influencing microtubule stability, axonal transport, cell-cycle regulation, and cytoskeletal organization. SIRT2 additionally acts on nuclear and metabolic substrates involved in oxidative stress, lipid metabolism, inflammation, proteostasis, and neuronal death. Consequently, pharmacological SIRT2 inhibition can increase the acetylation of downstream substrates and modify pathways associated with α-synuclein toxicity, ischemic injury, and neuroinflammation. Nevertheless, the effects of SIRT2 are context-dependent, because the enzyme may exert either detrimental or protective functions depending on the cell type, disease stage, and experimental model [21]. In several models of neurodegenerative disease, SIRT2 appears to contribute to neuronal damage. Pharmacological or genetic inhibition of SIRT2 reduced α-synuclein toxicity in cell-based models, primary neuronal cultures, and *Drosophila* models of Parkinson’s disease [22,23]. In primary mouse cortical neurons subjected to oxygen-glucose deprivation, an in vitro model of ischemic injury, pharmacological SIRT2 inhibition with AK-1 or AGK2 reduced apoptotic neuronal death and decreased pro-apoptotic signaling. In C57BL/6 mice subjected to middle cerebral artery occlusion followed by reperfusion, AGK2 additionally reduced infarct size and improved neurological outcomes, providing in vivo evidence for a detrimental role of SIRT2 during ischemic injury [24]. These findings suggest that excessive or abnormal SIRT2 activity may increase protein-related stress, apoptosis, and neuronal damage under pathological conditions [22,23].

However, other studies indicate that SIRT2 can also exert protective functions, especially in glial and immune contexts. In SIRT2-deficient mice subjected to intracortical lipopolysaccharide (LPS) administration, loss of SIRT2 enhanced microglial activation, pro-inflammatory cytokine production, nitrosative stress, and neuronal death. Complementary experiments in microglial models showed that SIRT2 overexpression suppressed Toll-like receptor-induced activation, whereas SIRT2 reduction increased inflammatory signaling and neurotoxicity, partly through regulation of nuclear factor kappa B (NF-κB) acetylation [25]. Consistent with this, systemic SIRT2 inhibition by compound i33 in a transgenic Alzheimer’s disease model improved cognition, long-term learning and memory, reduced cortical and hippocampal amyloid-β plaque burden, and central neuroinflammation (lowered brain expression of transforming growth factor beta, TGF-β and tumor necrosis factor alpha, TNF-α). In contrast, the same treatment increased peripheral inflammatory signaling, including interleukin IL-1β in white adipose tissue and elevated serum interleukin IL-6, monocyte chemoattractant protein-1 (MCP-1), and TNF-α, highlighting a marked divergence between central and systemic effects of SIRT2 inhibition. Moreover, peripheral SIRT2 inhibition with blood–brain barrier-impermeable AGK2 worsened cognitive performance and systemic inflammation [26]. In oligodendrocytes, SIRT2 is implicated in differentiation, myelination, and axonal metabolic support, suggesting that prolonged or nonselective inhibition could interfere with white-matter homeostasis. Recent studies therefore emphasize that therapeutic targeting of SIRT2 requires careful consideration of cell type, disease stage and treatment duration. Its inhibition may be beneficial in some disease models, but this effect is unlikely to be universal, especially when treatment is prolonged, poorly selective, or affects tissues outside the brain [27,28].

The mitochondrial isoform SIRT3 is considered the principal mitochondrial deacetylase. It regulates enzymes involved in the tricarboxylic acid cycle, fatty-acid oxidation, electron transport, ATP production, and antioxidant defense. For example, SIRT3-mediated deacetylation can enhance the activity of mitochondrial superoxide dismutase and other antioxidant enzymes, thereby limiting reactive oxygen species and maintaining mitochondrial integrity. In the brain, these effects are relevant to neuronal survival, metabolic adaptation, mitophagy, and resistance to oxidative injury [29].

In Alzheimer’s disease, reduced or dysregulated SIRT3 has been associated with mitochondrial dysfunction, p53-mediated neuronal injury, and impaired cerebral energy metabolism. Human postmortem evidence links SIRT3 dysregulation to mitochondrial dysfunction in Alzheimer’s disease. In the cerebral cortex of patients with Alzheimer’s disease, SIRT3 messenger RNA and protein levels were reduced, whereas mitochondrial acetylated p53 was increased. Further experiments showed that SIRT3 deacetylated p53 and attenuated p53-mediated repression of the mitochondrial DNA-encoded NADH dehydrogenase genes ND2 and ND4, thereby reducing reactive oxygen species and improving mitochondrial oxygen consumption. These findings provide human-tissue and cellular evidence that reduced SIRT3 activity is associated with mitochondrial dysfunction and neuronal injury in Alzheimer’s disease, although they do not establish therapeutic efficacy of SIRT3 activation in patients [30].

In Parkinson’s disease mouse models, SIRT3 appears to protect substantia nigra dopaminergic neurons from mitochondrial dysfunction and oxidative stress. Genetic deletion of Sirt3 increased mitochondrial oxidative stress and reduced mitochondrial membrane potential in these neurons. One important mechanism involves manganese superoxide dismutase (MnSOD), a major mitochondrial antioxidant enzyme that converts superoxide radicals into hydrogen peroxide and thereby limits oxidative damage. SIRT3 deacetylates MnSOD and enhances its antioxidant activity, whereas loss of SIRT3 is associated with increased MnSOD acetylation and reduced enzymatic activity. Thus, SIRT3 supports mitochondrial antioxidant defenses and may reduce the vulnerability of dopaminergic neurons to oxidative injury [31].

SIRT3 also participates in the response to cerebral ischemia and other acute brain injuries. Sirt3 deficiency aggravates neuronal apoptosis, neurological impairment, inflammatory responses, and blood–brain barrier disruption after ischemic stroke [32].

SIRT3 may additionally influence cognition and anxiety through regulation of hippocampal inhibitory neurotransmission. In male mice, intermittent fasting increased hippocampal GABA (gamma-aminobutyric acid)-mediated inhibitory neurotransmission, reduced anxiety-like behavior, and improved hippocampus-dependent memory. These adaptations were abolished in Sirt3-deficient mice and in wild-type mice with selective SIRT3 depletion in hippocampal neurons, indicating that neuronal SIRT3 is required for these effects. SIRT3 connects mitochondrial metabolism with neuronal-network plasticity rather than acting solely as an antioxidant enzyme [33].

Evidence in depression is emerging but remains predominantly experimental. Chronic-stress depression models have associated reduced SIRT3 signaling with mitochondrial oxidative stress and depressive-like behavior. Aerobic exercise increased hippocampal SIRT3 expression in chronically stressed rats [34], while the antidepressant and anxiolytic effects of kaempferol were partially lost in Sirt3-deficient mice, suggesting that SIRT3 contributes to the behavioral effects of some metabolic and antioxidant interventions [35]. Other compounds, such as α-cyperone and resveratrol, have also shown antidepressant-like effects in experimental models. These effects were accompanied by changes in SIRT3-related pathways involved in mitochondrial function and antioxidant defense [36,37].

More direct psychiatric evidence has emerged from models of early-life stress and schizophrenia-like behavior. Maternal separation of Wistar rat pups reduced NAD^+^ availability and SIRT3 expression in the prefrontal cortex and hippocampus, increased SOD2 (superoxide dismutase 2) acetylation and microglial inflammatory activation, and produced cognitive and schizophrenia-like behavioral abnormalities. Nicotinamide supplementation or treatment with honokiol reversed several of these changes, whereas SIRT3 inhibition attenuated the beneficial effects. The mentioned substances were administered after the early-life stress exposure (from postnatal day 56), indicating an important therapeutic rather than preventive intervention [38,39].

SIRT4 has relatively weak classical deacetylase activity and instead displays ADP-ribosyltransferase, lipoamidase/delipoylase, and several lysine deacylase activities. It exhibits ADP-ribosyltransferase, lipoamidase, and lysine deacylase activities and participates in the regulation of amino-acid metabolism, fatty-acid oxidation, insulin secretion, mitochondrial dynamics, and cellular stress responses. Its neurological functions remain less well characterized than those of SIRT1-SIRT3, although its regulation of mitochondrial metabolism suggests potential relevance to neuronal vulnerability and ageing [40].

Xing et al. reported that SIRT4 expression was increased in cellular and animal models of Alzheimer-like pathology and that SIRT4 promoted neuronal apoptosis and amyloid-β deposition. Inhibition or knockdown of SIRT4 reduced apoptotic signaling and pathological changes, whereas SIRT4 overexpression aggravated them. These findings suggest that excessive SIRT4 activity may impair neuronal survival, potentially through dysregulation of mTOR-dependent metabolic and survival pathways [41]. Other studies also showed that amyloid-β exposure can increase mitochondrial SIRT4 protein together with other mitochondrial stress-associated proteins. This observation supports the idea that SIRT4 is responsive to amyloidogenic stress, although it was not established whether the increase is initially adaptive or directly pathogenic [42].

SIRT5 is predominantly mitochondrial and functions mainly as a desuccinylase, demalonylase, and deglutarylase rather than as a conventional deacetylase. It regulates enzymes involved in the urea cycle, fatty-acid oxidation, glycolysis, mitochondrial respiration, and antioxidant defense. By controlling these less common lysine acyl modifications, SIRT5 contributes to metabolic flexibility and redox homeostasis, although its precise role in neurodegenerative and psychiatric disorders remains incompletely defined [43].

In neurological disorders, the effects of SIRT5 appear to be strongly dependent on the disease model, cell type, and pathological stage. In experimental subarachnoid hemorrhage, SIRT5-mediated desuccinylation was reported to preserve mitochondrial energy metabolism, reduce oxidative injury, and improve neurological outcomes, suggesting a protective function during acute brain injury [44]. In contrast, a study of ischemic stroke found that microglial SIRT5 promoted neuroinflammation and aggravated ischemic injury. Regarding mechanism of action, SIRT5 enhanced inflammatory microglial responses, whereas its genetic or pharmacological suppression reduced pro-inflammatory signaling. These apparently opposing findings indicate that neuronal or mitochondrial SIRT5 activity may support metabolic resilience, while excessive SIRT5 activity in activated microglia may intensify neuroinflammation [45].

Evidence in Alzheimer’s disease is similarly complex. Experimental modulation of SIRT5 has been associated with changes in amyloid-β production, neurotrophic signaling, and activation of microglia and astrocytes. In human SH-SY5Y neuroblastoma cells exposed to amyloid-β (Aβ), SIRT5 overexpression increased autophagic activity and reduced oxidative stress, apoptosis, and Aβ-associated cellular injury. Complementary experiments in APP695/PS1-dE9 transgenic mice, a murine model of Alzheimer-like amyloid pathology, showed that increased SIRT5 expression reduced cerebral Aβ accumulation, oxidative stress, and glial activation. These findings suggest a protective role for SIRT5; however, the evidence remains limited to preclinical models and has not yet been consistently replicated [46].

The role of SIRT5 in psychiatric disorders is considerably less studied than that of SIRT1 or SIRT3. Current hypotheses are based mainly on its regulation of mitochondrial ATP production, oxidative stress, and cellular metabolic flexibility, processes that are frequently disrupted in depression, anxiety, and other stress-related conditions. Studies have shown that SIRT5 may influence mood by maintaining cerebral energy reserves and limiting metabolic stress; however, direct behavioral studies, human biomarker data, and isoform-selective intervention studies remain scarce [47].

SIRT6 is a predominantly nuclear chromatin-associated enzyme with deacetylase, deacylase, and mono-ADP-ribosyltransferase activities. It regulates histone acetylation, DNA double-strand-break repair, telomere maintenance, glucose and lipid metabolism, inflammatory gene expression, and genomic stability. In neural tissue, SIRT6 has been implicated in brain development, mitochondrial function, ageing, and resistance to metabolic and oxidative stress. However, its actions may differ among neurons, astrocytes, and microglia and according to the disease context [48]. Brain-specific Sirt6 deficiency in mice caused progressive DNA damage, neuronal apoptosis, learning and memory deficits, and neurodegenerative changes [49]. In Aβ42-exposed cells, induction of SIRT6 reduced DNA damage and improved cell survival [50]. Conversely, astrocyte-specific Sirt6 deletion produced antidepressant- and anxiolytic-like effects in chronically stressed mice without impairing learning and memory. These behavioral effects were associated with altered purine metabolism in the medial prefrontal cortex, including increased astrocytic inosine and cyclic adenosine monophosphate (AMPc) [51]. Therefore, SIRT6 stimulation may be advantageous for neuronal protection and cognitive disorders, whereas carefully targeted inhibition in astrocytes can represent a preliminary strategy for stress-related mood and anxiety disorders.

SIRT7 is located mainly in the nucleolus and nucleus, where it regulates ribosomal RNA transcription, ribosome biogenesis, chromatin organization, RNA processing, stress adaptation, and genome stability. SIRT7 exhibits deacetylase and desuccinylase activities, with additional deacylase activity, including deglutarylation, reported. Compared with SIRT1 and SIRT2, its functions in the central nervous system are less extensively studied, but emerging evidence connects SIRT7 with cellular ageing, metabolic regulation, and maintenance of proteostatic and genomic integrity [52].

Overall, sirtuins act as metabolic sensors because their enzymatic activity depends on intracellular NAD^+^ availability. Changes in the NAD^+^/NADH balance during ageing, energetic stress, inflammation, or mitochondrial dysfunction can therefore alter sirtuin activity and the acetylation or acylation state of their substrates. Through this connection, sirtuins coordinate cellular metabolism with epigenetic regulation, stress resistance, synaptic function, and survival [21]. Their effects are nevertheless isoform-, substrate-, cell-, and disease-specific; consequently, activation of one sirtuin may be beneficial in a particular disorder, whereas inhibition of another isoform, or even inhibition of the same isoform in a different biological context, may produce a more favorable outcome (Table 1).

Importantly, changes in total cellular NAD^+^ levels should not be interpreted as producing a uniform change in the activity of all sirtuin isoforms. NAD^+^ metabolism is compartmentalized across the nucleus, cytosol, and mitochondria, and local availability is determined by salvage and biosynthetic pathways, including nicotinamide phosphoribosyltransferase (NAMPT)-dependent recycling, as well as by NAD^+^ consumption through enzymes such as CD38 and poly(ADP-ribose) polymerases. In addition, the NADH/NAD^+^ redox balance and differences in subcellular localization may influence individual sirtuin responses. Consequently, NAD^+^ depletion or restoration may affect sirtuin isoforms differently depending on cellular compartment, metabolic state, and competing NAD^+^-consuming pathways.

## 5. Pharmacological Modulation of Sirtuins in Neurodegenerative and Psychiatric Disorders

**Figure 9:**
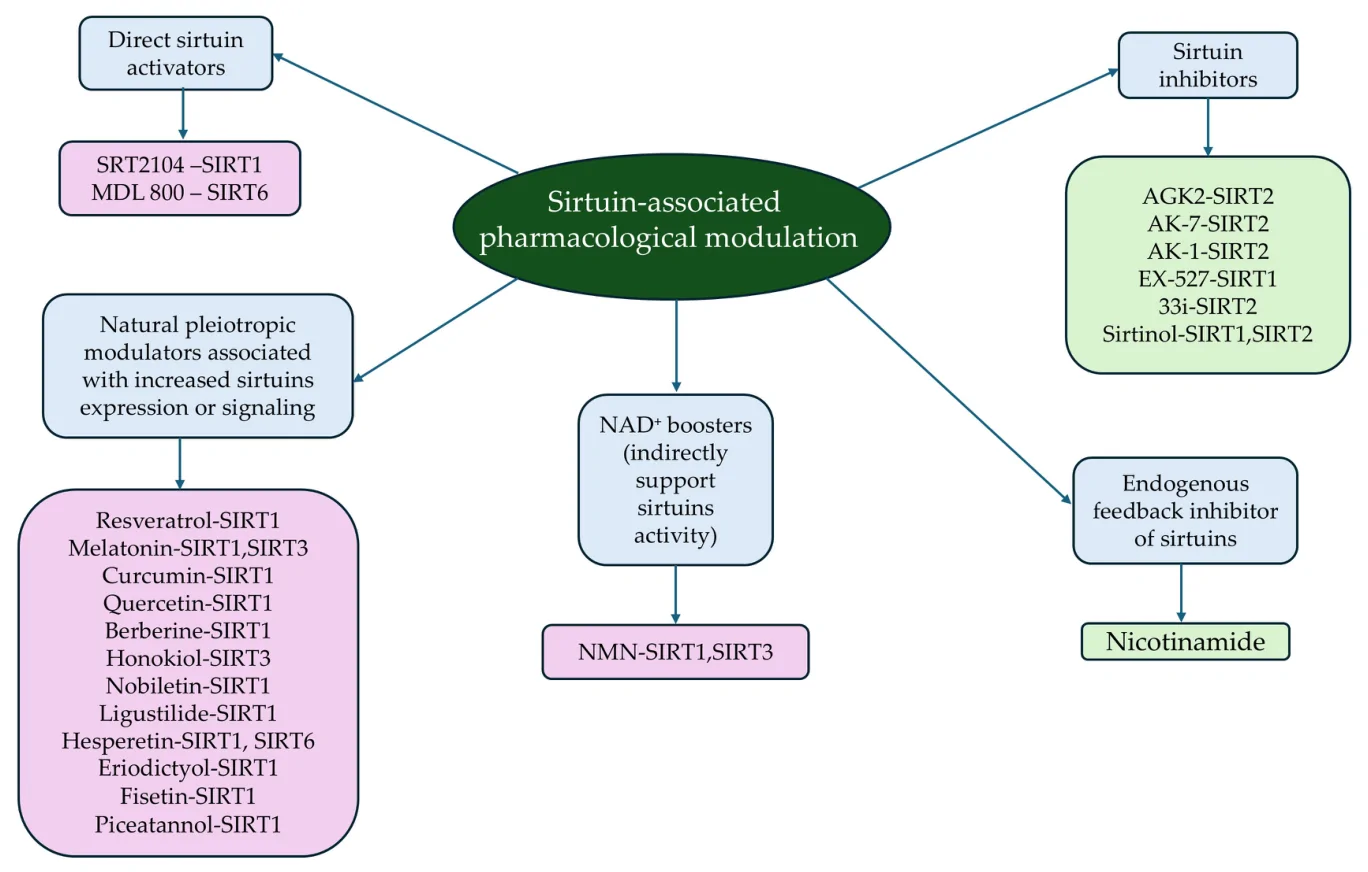
Classification of sirtuin-associated pharmacological modulators in neurological and psychiatric disorders.

This section evaluates pharmacological compounds that modulate sirtuin-associated pathways. Particular attention is given to isoform specificity, activation versus inhibition, direct versus indirect mechanisms, dose- and timing-dependent effects, preclinical versus clinical evidence, and translational limitations. To provide a conceptual framework for this discussion, the compounds reviewed in this section were organized into five pharmacological categories: direct or targeted sirtuin activators, natural pleiotropic modulators associated with increased sirtuin expression or signaling, NAD^+^ boosters that indirectly support sirtuin activity, synthetic sirtuin inhibitors, and context-dependent endogenous feedback inhibitors (Figure 9). This classification emphasizes that not all compounds described as sirtuin modulators act through the same mechanism, and that many natural compounds influence sirtuin-associated pathways indirectly rather than functioning as selective enzymatic activators.

For consistency, the term “activation” is used when there is evidence for a direct increase in sirtuin catalytic activity, whereas “enhancement” or “upregulation” refers to increased sirtuin expression, activity, or downstream signaling without necessarily implying direct enzyme activation. “Inhibition” denotes a demonstrated reduction in sirtuin catalytic activity, while “modulation” is used as a broader term when the direction or mechanism is context-dependent or incompletely defined. Compounds acting through upstream pathways, NAD^+^ metabolism, or downstream signaling are described as indirect or sirtuin-associated modulators.

### 5.1. Natural Pleiotropic Modulators of Sirtuins

Resveratrol is one of the most extensively investigated natural compounds proposed to modulate SIRT1 activity. It is a polyphenolic stilbene commonly associated with antioxidant, anti-inflammatory, metabolic, vascular-protective, and anti-aging effects [53]. In the context of sirtuin biology, resveratrol has frequently been described as a SIRT1 activator; however, its pharmacological profile remains complex and not fully specific. Resveratrol has been proposed to activate SIRT1 indirectly by increasing intracellular NAD^+^ availability. This may occur through activation of AMP-activated protein kinase and subsequent stimulation of NAD^+^ biosynthesis via nicotinamide phosphoribosyltransferase, thereby enhancing NAD^+^-dependent SIRT1 deacetylase activity [54,55]. However, the clinical relevance of this mechanism remains uncertain. The systematic review and dose–response meta-analysis by Mansouri et al. evaluated randomized controlled trials assessing the effect of oral resveratrol supplementation on human Sirtuin 1. The analysis included 11 randomized controlled trials published between 2011 and 2023, with a total of 632 adult participants, intervention durations ranging from 4 to 24 weeks, and doses ranging from 40 to 3000 mg/day. The dose–response meta-analysis of randomized controlled trials showed resveratrol did not significantly affect SIRT1 gene expression in the overall pooled analysis; however, subgroup analyses suggested a significant increase in SIRT1 gene expression in trials lasting less than 12 weeks and in studies assessing blood-derived tissues [56]. In patients with type 2 diabetes, Bo et al. used 40 mg/day or 500 mg/day resveratrol for 6 months and found that SIRT1 levels were significantly higher in the 500 mg/day group at trial end, supporting a possible dose-related effect. However, the same study also noted that not all patients receiving resveratrol showed increased SIRT1 expression or activation, reinforcing the idea of interindividual variability [57]. Importantly, resveratrol has also been evaluated directly in neurological disease. In a 52-week randomized, double-blind, placebo-controlled phase II trial involving 119 patients with mild-to-moderate Alzheimer’s disease, resveratrol and its metabolites were detected in cerebrospinal fluid, providing evidence of central nervous system exposure; however, the study did not establish a clear clinical benefit on cognitive or functional outcomes [58]. In a retrospective analysis of biomarker-confirmed participants from the same phase II trial, resveratrol treatment for 52 weeks was associated with a marked reduction in cerebrospinal fluid matrix metalloproteinase-9 (MMP-9), together with changes in inflammatory and adaptive immune markers, including increased macrophage-derived chemokine, interleukin-4, and fibroblast growth factor-2. In the same subgroup, Mini-Mental State Examination (MMSE) scores declined significantly in the placebo group but not in the resveratrol group, while the reduction in Alzheimer’s disease cooperative study–activities of daily living (ADCS-ADL) scores was approximately twofold greater with placebo. Resveratrol also attenuated the decline in cerebrospinal fluid amyloid-β42, whereas tau levels were unchanged. Because these findings were obtained from a retrospective analysis of a small biomarker-defined subgroup, they should be regarded as supportive but exploratory rather than as definitive evidence of clinical efficacy [59]. Resveratrol remains the best-known natural compound associated with SIRT1 activation, but current clinical evidence indicates that its effects are inconsistent, dose- and duration-dependent, and strongly influenced by tissue type and biological context. Therefore, in the context of neuropsychiatric disorders, resveratrol should be discussed not as a selective SIRT1 agonist, but as a pleiotropic compound with metabolic, antioxidant, anti-inflammatory, and epigenetic effects, among which SIRT1 modulation represents only one possible mechanism.

Eriocitrin, or eriodictyol 7-O-rutinoside, is a citrus-derived flavanone glycoside found mainly in lemons and related citrus products. After oral intake, it is extensively metabolized by the gut microbiota to eriodictyol, which is subsequently absorbed and converted through glucuronidation, sulfation, and methylation into circulating metabolites such as hesperetin, homoeriodictyol, and their conjugates. Consequently, the biological effects of orally administered eriocitrin are likely mediated by both the parent compound and its multiple metabolites [60]. The aglycones eriodictyol, hesperetin, and homoeriodictyol are smaller and more lipophilic than the glycosides eriocitrin and hesperidin and more likely to exhibit improved systemic bioavailability and blood–brain barrier penetration [61].

Because eriocitrin is extensively metabolized after oral administration, its neuroprotective effects may partly reflect the activity of its major metabolites, particularly eriodictyol and hesperetin. These compounds have been shown to modulate distinct sirtuin-associated pathways relevant to neuroinflammation and cognitive decline. Among eriocitrin-derived metabolites, eriodictyol has shown particularly promising neuroprotective effects in experimental models of Alzheimer’s disease. Thus, eriodictyol exerts neuroprotective effects through coordinated antioxidant, anti-inflammatory, anti-apoptotic, and cholinergic mechanisms. In neuronal models exposed to H_2_O_2_ or Aβ25–35, pre-treatment with eriodictyol activates the Nrf2/antioxidant response element pathway, increases antioxidant protection, preserves mitochondrial membrane potential, suppresses JNK/p38 MAPK signaling, and reduces caspase-mediated apoptosis [62,63,64]. In LPS-induced models of neuroinflammation, including both cultured cells and experimental animals, pre-treatment with eriodictyol reduced inflammatory signaling and improved cognitive and neuronal outcomes. It limits Aβ accumulation, inhibits acetylcholinesterase, and restores choline acetyltransferase activity and acetylcholine levels, thereby simultaneously targeting oxidative stress, neuroinflammation, amyloidogenesis, mitochondrial dysfunction, and cholinergic impairment [61,65,66]. Eriodictyol attenuated LPS-induced synaptic impairment by increasing SIRT1 expression, while simultaneously suppressing NF-κB- and MAPK-related inflammatory signaling and improving cognitive performance, suggesting the involvement of a SIRT1-linked anti-inflammatory and synaptic-protective mechanism [65].

Similarly, in H_2_O_2_-exposed SH-SY5Y cells, pre-treatment with hesperetin (10–40 μM) enhanced neuronal survival, reduced reactive oxygen species (ROS) production and inflammatory signaling, and limited apoptosis. These effects were accompanied by increased SIRT1 signaling and AMPK phosphorylation, together with reduced mTOR phosphorylation. This coordinated response may facilitate the clearance of damaged cellular components and preserve neuronal homeostasis under oxidative stress [67]. Beyond SIRT1, hesperetin has also been associated with SIRT6 regulation. In scopolamine-induced cognitive impairment, hesperetin increased SIRT6 expression and inhibited the TXNIP/NLRP3 inflammasome pathway, resulting in reduced oxidative stress, inflammatory cytokine production, and neuronal injury, together with restoration of cholinergic neurotransmission and improved learning and memory [68].

Nobiletin is a naturally occurring polymethoxylated flavone, chemically identified as 5,6,7,8,3′,4′-hexamethoxyflavone, and is particularly enriched in citrus peel. Its high degree of methoxylation distinguishes it from many other citrus flavonoids and is generally associated with increased lipophilicity and potentially improved tissue penetration. Nobiletin has been investigated as an indirect modulator of SIRT1 signaling in experimental Alzheimer. In a streptozotocin-induced mouse model of Alzheimer-like pathology, nobiletin was administered at 50 mg/kg/day for 21 days, either alone or in combination with EX-527, a selective SIRT1 inhibitor, to determine whether its neuroprotective effects were mediated through SIRT1 signaling. Nobiletin markedly restored hippocampal SIRT1 protein expression, which had been substantially reduced by streptozotocin. These findings suggest that nobiletin restores SIRT1/FoxO3a-related autophagy. Through this pathway, SIRT1 may activate FoxO3a-dependent autophagy genes, helping neurons to remove damaged proteins and dysfunctional organelles. This mechanism is particularly relevant to Alzheimer’s disease, in which impaired autophagy contributes to the accumulation of amyloid-β, tau pathologic protein, dysfunctional mitochondria, and other neurotoxic substrates [69].

Pharmacokinetic evidence further supports the ability of nobiletin to reach the central nervous system in preclinical models. Following a single oral dose of 50 mg/kg in rats, nobiletin reached its maximum concentration after 1 h in both plasma and brain, with a higher maximum concentration in brain (4.20 μg/mL) than in plasma (1.78 μg/mL). The elimination half-life was substantially longer in brain than in plasma, providing direct evidence of sustained cerebral exposure in rodents [70]. Human evidence is more limited. In a multicenter, randomized, double-blind, placebo-controlled study in healthy older adults, 16-week administration of a nobiletin-rich formulation was associated with improvements in general and visual memory compared with placebo; however, the intervention contained additional botanical components and therefore does not establish the direct efficacy of isolated nobiletin [71].

Ligustilide is a naturally occurring, lipophilic phthalide derivative and a major bioactive constituent of *Angelica sinensis* and *Ligusticum chuanxiong*, investigated for its antioxidant, anti-inflammatory, and neuroprotective properties [72]. Following oral administration in rats, ligustilide was detected in multiple tissues, with relatively high concentrations in the cerebrum and cerebellum, supporting brain distribution [73]. Vascular dementia is often associated with reduced SIRT1 expression and activation of the endoplasmic-reticulum stress pathway. In a rat model of vascular dementia, oral treatment with ligustilide at 20 or 40 mg/kg improved learning and memory, with the higher dose generally producing more pronounced effects. These cognitive benefits were accompanied by reduced neuronal degeneration, oxidative stress, endoplasmic-reticulum stress, and apoptosis [74].

Honokiol, a bioactive biphenolic compound derived from *Magnolia* species, is one of the natural compounds most consistently linked to SIRT3-associated neuroprotection. Honokiol has strong preclinical evidence of central nervous system exposure. In rats, honokiol crossed both the blood–brain barrier and the blood-cerebrospinal fluid barrier, with brain-to-plasma concentration ratios exceeding unity and increasing to approximately 2.5–2.7 at later sampling times after intravenous administration [75]. In Alzheimer-like cellular models, honokiol increased SIRT3 expression, improved mitochondrial function, reduced reactive oxygen species and lipid peroxidation, enhanced antioxidant defenses, and attenuated amyloidogenic processing, including reductions in Aβ levels [76]. In APP/PS1 mice, honokiol improved cognitive impairment and synaptic damage by promoting mitochondrial quality-control mechanisms, particularly SIRT3-dependent mitophagy and activation of the mitochondrial unfolded-protein response [77]. More recent work extended this mechanism to neuronal ferroptosis, showing that pretreatment with honokiol attenuated anesthesia/surgery-induced cognitive impairment by increasing SIRT3 expression and reducing mitochondrial GPX4 (glutathione peroxidase 4) acetylation, thereby limiting ferroptotic neuronal injury [78]. Honokiol-induced SIRT3 upregulation has also been implicated in hippocampal protection after pilocarpine-induced status epilepticus. In this context, post-treatment with honokiol increased SIRT3 expression and reduced glial activation, including reactive astrocytosis and microglial activation, suggesting that SIRT3-associated signaling may contribute to anti-inflammatory neuroprotection after severe epileptic injury [79]. In psychiatric models, honokiol has shown antidepressant- and anxiolytic-like effects, but these effects have not been consistently linked to SIRT3 or other sirtuin isoforms. In an LPS-induced depression model, pretreatment with honokiol improved depression-like behavior by suppressing hippocampal NF-κB activation, reducing inflammatory signaling, lowering quinolinic acid accumulation, and reducing calcium overload [80].

Fisetin is a flavonol structurally related to quercetin and is found in strawberries, apples, persimmons, onions, and cucumbers. It is often discussed as both a neuroprotective flavonoid and a senolytic compound, but its sirtuin-related central nervous system evidence is again centered mainly on SIRT1-associated pathways. In a D-galactose-induced brain ageing model, fisetin, administered post-induction, reduced oxidative stress, neuroinflammation, synaptic dysfunction, and memory impairment, while regulating the SIRT1/Nrf2/HO-1 pathway in mouse brain [81]. In psychiatric models, fisetin has antidepressant-like activity, but the mechanism is not documented to be sirtuin-specific. Early work showed that fisetin produced antidepressant-like effects in mouse behavioral despair tests through serotonergic and noradrenergic systems [82].

Piceatannol is a hydroxylated analogue and metabolite of resveratrol, belonging to the stilbene family. Piceatannol has recent evidence for SIRT1-dependent neuroprotection in stress-related and ischemic models. In cerebral ischemia/reperfusion injury, post-treatment with piceatannol improved neurological function and reduced neuronal apoptosis and oxidative stress through the SIRT1/FoxO1 pathway; inhibition of this pathway reversed the antioxidant effects of treatment, supporting SIRT1 pathway involvement [83]. Piceatannol has also been investigated in chronic unpredictable stress-induced neurobehavioral deficits. In this model, piceatannol attenuated anxiety/depression-like behaviors and cognitive impairment, while reducing neuroinflammation, oxidative stress, and neurotransmitter imbalance through SIRT1-mediated mechanisms [84]. A related report found that the neuroprotective effects of piceatannol in chronic unpredictable stress-induced memory dysfunction were abolished by pretreatment with sirtinol, a SIRT1 inhibitor, strengthening the interpretation that SIRT1 contributes to its protective effect [85].

Berberine is a naturally occurring isoquinoline alkaloid found in several medicinal plants, including *Berberis* species, *Coptis chinensis*, and *Hydrastis canadensis*. In the nervous system, its protective effects have been linked to antioxidant, anti-inflammatory, anti-apoptotic, mitochondrial, and autophagy-regulating mechanisms. These effects were associated with modulation of the AMPK–PGC-1α–SIRT1 axis, suggesting that berberine may enhance SIRT1-related mitochondrial protection. The study involved 6-OHDA-treated SH-SY5Y cells and a 6-OHDA-induced Parkinson’s disease mouse model, and the authors reported that berberine reduced oxidative stress, restored mitochondrial function, improved autophagy-related markers, and modulated AMPK phosphorylation together with SIRT1 and PGC-1α protein expression [86]. However, another study showed that berberine did not protect dopaminergic-like PC12 cells from 6-OHDA toxicity. Instead, berberine at 10 and 30 μM enhanced 6-OHDA-induced cytotoxicity after 48 h, with increased apoptotic cell death compared with 6-OHDA alone. The same study also reported that berberine treatment in unilateral 6-OHDA-treated rats worsened dopaminergic degeneration and dopamine depletion in the substantia nigra, suggesting that under those experimental conditions berberine aggravated rather than attenuated Parkinson-like neurotoxicity [87]. This contrasts with more recent work reporting protective effects of berberine in 6-OHDA models through mitochondrial and autophagy-related pathways. The discrepancy likely reflects differences in cell model, differentiation status, dose, treatment timing, duration of exposure, and 6-OHDA concentration. For example, some studies report hormetic effects, where low-dose berberine can activate adaptive survival pathways such as PI3K/Akt/Bcl-2 and Nrf2/HO-1, whereas higher concentrations or prolonged exposure may become cytotoxic or amplify oxidative injury [88].

Melatonin is an indoleamine primarily secreted by the pineal gland and widely known for regulating circadian rhythms, but it also has potent antioxidant, anti-inflammatory, anti-apoptotic, mitochondrial-protective, and immunomodulatory actions. In neurological models, these properties intersect with sirtuin signaling because melatonin can increase SIRT1 or SIRT3 expression/activity-related pathways, thereby promoting antioxidant defense, mitochondrial homeostasis, autophagy regulation, and suppression of neuroinflammation. In an LPS-induced neuroinflammation and depression-like behavior mouse model, pre-treatment with melatonin improved behavioral abnormalities, reduced hippocampal cytokine production, oxidative stress, and inflammatory signaling, and normalized the LPS-induced disruption of SIRT1, Nrf2, and HO-1 expression. This supports a SIRT1/Nrf2/HO-1-associated antidepressant and anti-inflammatory mechanism [89]. A related study showed that pre-treatment with melatonin attenuated LPS-induced acute depressive-like behaviors and microglial NLRP3 inflammasome activation through the SIRT1/Nrf2 pathway; importantly, the protective effects were reversed by SIRT1 inhibition and Nrf2 silencing, strengthening the mechanistic link to SIRT1-dependent signaling [90]. In a postnatal rat model of LPS-induced neuroinflammation, melatonin administered after LPS exposure protected the hippocampal dentate gyrus against oxidative stress, acute neuroinflammation, and apoptotic neurodegeneration through activation of the SIRT1/Nrf2 pathway [91]. Beyond SIRT1, melatonin has evidence for SIRT3-associated mitochondrial protection. In diabetic mice subjected to focal cerebral ischemia–reperfusion injury, melatonin administered intraperitoneally at 20 mg/kg after ischemia and before reperfusion reduced infarct volume, neurological deficits, and brain edema, while improving mitochondrial function and reducing mitochondrial oxidative damage. These effects were mediated at least partly through activation of the Akt-SIRT3-SOD2 pathway, because inhibition of Akt weakened melatonin-induced SIRT3 upregulation and reduction in SOD2 acetylation. This finding is important because it connects melatonin to mitochondrial sirtuin-dependent antioxidant defense [92]. In a randomized, double-blind, placebo-controlled trial involving 60 patients with Parkinson’s disease, melatonin supplementation at 10 mg/day for 12 weeks improved several non-motor outcomes, like sleep quality, depression, and anxiety scores compared with placebo [93]. However, the study did not assess SIRT1 or SIRT3 target engagement, and therefore does not establish that the observed clinical effects were mediated through sirtuin signaling.

Curcumin, the principal polyphenolic constituent of *Curcuma longa*, is a pleiotropic natural compound with antioxidant, anti-inflammatory, anti-apoptotic, mitochondrial-protective, autophagy-modulating, and anti-amyloid properties. Human pharmacokinetic studies demonstrate very low systemic exposure to native oral curcumin, whereas micronized and particularly micellar formulations substantially increase bioavailability [94]. Its relationship with sirtuin biology is mainly centered on SIRT1-associated signaling. In Aβ25–35-exposed rat cortical neurons, pre-treatment with curcumin attenuated neuronal death, oxidative stress, mitochondrial dysfunction, and apoptosis, with SIRT1 activation implicated in the protective response [95]. In cerebral ischemia models, curcumin has also been linked to SIRT1-associated protection. A study on experimental stroke reported that curcumin pretreatment attenuated inflammation and mitochondrial dysfunction after cerebral ischemia/reperfusion injury, with SIRT1 signaling proposed as a relevant mechanism [96]. Reviews of curcumin in cerebral ischemia also identify SIRT1 among the pathways involved in its anti-inflammatory and neuroprotective effects. Curcumin has documented antidepressant- and anxiolytic-like effects in experimental and clinical literature, while in Parkinson’s disease models, curcumin has shown neuroprotective effects; however, these effects have not yet been consistently demonstrated to depend on sirtuin signaling [97].

Quercetin is a naturally occurring dietary flavonol, and it has been widely investigated for antioxidant, anti-inflammatory, anti-apoptotic, metabolic, and neuroprotective properties. In the context of sirtuin biology, its most relevant evidence concerns SIRT1-associated signaling, especially in diabetic encephalopathy and cognitive impairment. A key study investigated quercetin in db/db mice, a type 2 diabetes model associated with diabetic encephalopathy. Quercetin improved cognitive performance and reduced neuronal injury in the hippocampus. These effects were associated with increased SIRT1 expression and inhibition of endoplasmic-reticulum stress markers. Quercetin also suppressed NLRP3 inflammasome activation, suggesting that SIRT1-associated inhibition of neuroinflammation may contribute to its protective effects [98]. Clinical evidence of quercetin’s effects remains limited and heterogeneous. In a small randomized, double-blind, placebo-controlled study involving patients with mild cognitive impairment or Alzheimer’s disease, continuous intake of quercetin-rich onion powder was associated with changes suggestive of improved motivated or emotional behavior [99]. More recently, a randomized, triple-blind, placebo-controlled trial in adults with autism spectrum disorder reported a longitudinal reduction in social responsiveness scale scores during quercetin treatment [100]. However, human SIRT1 target engagement remains unestablished.

### 5.2. Direct Sirtuin Activators

MDL-800 is a direct small-molecule sirtuin activator, and it was specifically identified as a selective allosteric activator of SIRT6 rather than merely as a compound that increases sirtuin expression. Huang et al. reported that MDL-800 increased SIRT6 deacetylase activity by up to 22-fold through binding to an allosteric site [101]. Its relevance to neurological disease has recently been extended to vascular cognitive impairment. In a mouse model of chronic cerebral hypoperfusion induced by bilateral common carotid artery stenosis, neuronal SIRT6 protein expression was reduced, and neuron-specific Sirt6 deletion aggravated neuronal damage and cognitive deficits. Conversely, post-treatment with MDL-800 mitigated neuronal loss and improved neurological and cognitive recovery, supporting the therapeutic potential of SIRT6 activation in hypoperfusion-related cognitive impairment [102]. These findings support SIRT6 activation as a potential strategy for vascular cognitive impairment, although the evidence remains preclinical and direct clinical validation of MDL-800 in neuropsychiatric disorders is still lacking.

SRT2104 is a synthetic small-molecule SIRT1 activator belonging to the second generation of sirtuin-activating compounds, developed from optimization of the imidazo-thiazole scaffold. SRT2104 contains an imidazo-thiazole core, a thiazole-carboxamide moiety, a pyridinyl substituent, and a morpholinylmethyl group, structural features that support its interaction with the hydrophobic pocket of SIRT1. Docking analyses suggest that SRT2104 is stabilized within the SIRT1 binding pocket through multiple non-covalent interactions [103]. Human phase I studies evaluated oral SRT2104 over a dose range of 0.03–3.0 g in healthy volunteers and showed acceptable short-term tolerability with dose-dependent systemic exposure. However, human pharmacokinetic studies revealed relatively low oral bioavailability (~14%), substantial interindividual variability, sub-proportional exposure with increasing dose, and a marked food effect. Moreover, although systemic pharmacokinetics and short-term tolerability have been characterized in humans, human brain exposure and neurological efficacy have not been established [104]. In preclinical models, SRT2104 has shown neuroprotective, neuroplasticity-related, and antidepressant-like effects. In Huntington’s disease mice, SRT2104 was administered chronically in the diet at 0.5% starting at 6 weeks of age and continued until the end of life. This early disease-modifying treatment penetrated the blood–brain barrier, improved motor performance, attenuated brain atrophy, and extended survival [105]. In depression-related models, SRT2104 has also been tested after chronic stress exposure. In chronic unpredictable mild stress mice, hippocampal SRT2104 administration reversed depressive-like behavior, restored stress-reduced hippocampal SIRT1 expression [106]. An additional mouse model of depression showed that chronic stress reduced SIRT1 activity in the hippocampal dentate gyrus, whereas SRT2104 treatment prevented stress-induced dendritic spine loss, supporting a role for SIRT1 activation in stress-related synaptic plasticity [107]. In diabetes-related cognitive dysfunction, intracerebroventricular administration of SRT2104 to streptozotocin-induced diabetic rats with cognitive impairment increased hippocampal SIRT1 and brain-derived neurotrophic factor (BDNF) expression, suggesting that SIRT1 activation may improve cognitive dysfunction by restoring hippocampal neurotrophic and synaptic signaling [108].

SRT2104 is a synthetic SIRT1-activating compound with a more defined pharmacological profile than pleiotropic natural modulators such as resveratrol. In N171-82Q Huntington-like mice, chronic dietary administration resulted in measurable brain concentrations and improved motor performance, reduced brain atrophy, and prolonged survival, demonstrating CNS exposure and preclinical efficacy. However, human pharmacokinetic studies revealed relatively low oral bioavailability (~14%), substantial interindividual variability, sub-proportional exposure with increasing dose, and a marked food effect. Moreover, although systemic pharmacokinetics and short-term tolerability have been characterized in humans, human brain exposure and neurological efficacy have not been established [104]. Thus, SRT2104 should be considered a comparatively selective experimental SIRT1 modulator with human systemic pharmacokinetic data rather than a clinically validated CNS therapy.

### 5.3. NAD^+^ Boosters

NAD^+^-boosting strategies should be regarded as metabolic interventions that may indirectly influence sirtuin activity rather than as equivalent to selective activation of a specific sirtuin isoform.

Nicotinamide mononucleotide (NMN) is an endogenous nucleotide and immediate precursor of nicotinamide adenine dinucleotide (NAD^+^), a key metabolic cofactor required for mitochondrial bioenergetics, redox homeostasis, DNA repair, and sirtuin enzymatic activity. By increasing intracellular NAD^+^ availability, NMN is considered an indirect sirtuin-supporting intervention rather than a direct sirtuin activator. NMN protected against Aβ oligomer-induced neuronal death and cognitive impairment in hippocampal slice cultures and a rat Alzheimer disease model [109]. A single dose of NMN, 62.5 mg/kg, increased hippocampal mitochondrial NAD^+^ pools for up to 24 h in male mice and produced a SIRT3-mediated decrease in mitochondrial protein acetylation [110]. In a corticosterone-induced depression-like mouse model, NMN alleviated depression-like behavior and improved disrupted mitochondrial bioenergetics; importantly, Sirt3 knockdown in primary mouse astrocytes reversed the effect of NMN on energy production, supporting involvement of SIRT3 [111]. For acute brain injury, a recent post-cardiac arrest study showed that systemic NMN administration improved neurological function, increased 7-day survival, reduced hippocampal neuronal injury, restored brain NAD^+^ and ATP levels, and increased brain SIRT3 levels after cardiac arrest [112].

### 5.4. Sirtuin Inhibitors

AGK2 is a synthetic, cell-permeable small molecule used primarily as a pharmacological inhibitor of SIRT2, a cytosolic and partly nuclear NAD^+^-dependent deacetylase. AGK2 inhibits SIRT2 with a reported half-maximal inhibitory concentration of approximately 3.5 μM, while substantially higher concentrations are required to inhibit SIRT1 or SIRT3. It should therefore be described as a relatively selective, but not absolutely isoform-specific, SIRT2 inhibitor [23]. Pharmacological inhibition of SIRT2 by AGK2 increases the acetylation state of SIRT2 substrates and alters downstream signaling rather than decreasing SIRT2 protein expression [113]. In neuronal cultures and *Drosophila* models of Parkinson-like pathology, pharmacological or genetic inhibition of SIRT2 protected dopaminergic neurons and modified α-synuclein inclusion morphology. SIRT2 inhibition appears to favor the formation of larger, less diffusely distributed α-synuclein inclusions, which may sequester toxic soluble or oligomeric species. Consequently, AGK2 may protect dopaminergic neurons not simply by reducing total α-synuclein, but by altering its aggregation state [23].

EX-527 (selisistat), a potent and selective SIRT1 inhibitor, has been shown to promote the differentiation of pluripotent P19 cells into functionally mature neuron-like cells. EX-527 inhibits human SIRT1 in the nanomolar range and displays more than 200-fold selectivity over SIRT2 and SIRT3; structural and kinetic studies indicate that its inhibitory mechanism is dependent on the NAD^+^-coupled catalytic cycle [114,115]. Pharmacological inhibition of SIRT1 enhanced neuronal marker expression, morphological differentiation, and electrophysiological maturation, suggesting that SIRT1 can restrain neuronal lineage commitment in pluripotent cells. These findings emphasize the context-dependent role of SIRT1: while its activation may support survival and stress resistance in mature neurons, its inhibition can facilitate neuronal differentiation in progenitor-like cellular systems [116]. In Huntington’s disease mice, orally administered EX-527 achieved brain concentrations approximately twice those measured in plasma and improved survival and selected locomotor outcomes, supporting both brain penetration and preclinical efficacy [117]. A 12-week phase II study involving 144 patients similarly provided safety and pharmacokinetic data; reversible elevations in liver-function tests occurred in a subset of treated participants [118].

AK-7 is a brain-permeable SIRT2 inhibitor developed to improve the pharmacological profile of earlier SIRT2 inhibitors such as AGK2. In differentiated human LUHMES neuronal cells overexpressing α-synuclein, AK-7 reduced α-synuclein-associated neurotoxicity. Complementary, in vivo experiments in mice subjected to induced Parkinson-like neurodegeneration showed that systemic AK-7 treatment attenuated striatal dopamine depletion, preserved tyrosine hydroxylase-positive dopaminergic neurons in the substantia nigra, and improved motor performance. These findings provide preclinical evidence that SIRT2 inhibition can protect dopaminergic neurons in specific cellular and toxin-induced Parkinson-like models. In contrast, chronic AK-7 treatment did not alter disease onset or survival in SOD1-G93A transgenic mice, a genetic model of amyotrophic lateral sclerosis, and failed to reduce infarct volume, ischemic tissue swelling, neurological deficits, or mortality in an experimental mouse model of cerebral ischemia. These negative findings indicate that the neuroprotective effects of SIRT2 inhibition with AK-7 are strongly model-dependent [119]. Moreover, in experimental traumatic brain injury, AK-7, administered 30 min before injury, exacerbated neuroinflammation and blood–brain barrier disruption through increased acetylation and activation of NF-κB p65. These findings indicate that SIRT2 inhibition requires careful consideration of disease context, target tissue, timing, and immune consequences [120].

AK-1 is an early SIRT2 inhibitor used alongside AGK2 to probe the role of SIRT2 in neurodegenerative and ischemic models. In primary striatal neurons expressing mutant huntingtin and in *Drosophila* and *Caenorhabditis elegans* Huntington models, SIRT2 inhibition by AK-1 or AGK2 reduced toxicity, partly through modulation of sterol-biosynthesis pathways [121]. In primary cortical neurons subjected to oxygen-glucose deprivation, AK-1 reduced apoptotic neuronal death and pro-apoptotic signaling, supporting a protective effect of SIRT2 inhibition under ischemic conditions [24].

Compound 33i is a selective SIRT2 inhibitor evaluated in a transgenic Alzheimer’s disease mouse model. This compound improved cognitive dysfunction and long-term potentiation, reducing amyloid pathology and neuroinflammation in the APP/PS1 Alzheimer disease mouse model. However, treatment was also associated with increased peripheral inflammatory cytokine levels. This finding highlights an important translational limitation of SIRT2 inhibition, as beneficial effects within the central nervous system may coexist with adverse inflammatory responses in peripheral tissues [26]. Compound 33i has a more favorable pharmacological profile than earlier SIRT2 inhibitors. This 2-anilinobenzamide derivative inhibits SIRT2 with submicromolar potency and displays markedly greater isoform selectivity than AGK2 [122]. Functional central exposure following systemic administration is supported by pharmacodynamic studies showing SIRT2 inhibition and alterations in glutamatergic and serotonergic signaling within the mouse prefrontal cortex. Chronic 33i administration also reversed anhedonia and social avoidance in a chronic mild stress model, supporting antidepressant-like activity [123]. In the SAMP8 mouse model of age-related cognitive impairment, early systemic treatment improved spatial learning and memory and reduced neuroinflammation, whereas treatment initiated at a more advanced disease stage failed to reverse established cognitive decline, indicating stage-dependent efficacy [124].

Sirtinol is a nonselective SIRT1/SIRT2 inhibitor that is mainly used as an experimental probe of sirtuin-dependent signaling. In a chronic variable stress rat model, intra-dentate gyrus infusion of sirtinol prevented stress-induced memory impairment and modulated hippocampal ERK (extracellular signal-regulated kinase) signaling and histone acetylation [125].

Nicotinamide, also known as niacinamide, is the amide form of vitamin B3 and a product of NAD^+^-consuming reactions. It has a complex activity because it acts both as an NAD^+^ precursor/metabolic substrate and as an end-product inhibitor of sirtuins through feedback inhibition. In Alzheimer’s disease, nicotinamide restored cognitive deficits in 3xTg mice and selectively reduced Thr231-phosphorylated tau; these effects involved sirtuin inhibition, especially inhibition of SIRT2-like tubulin deacetylase activity [126]. In depression models, however, the interpretation is different: nicotinamide improved restraint stress-induced depressive-like behavior, but the study concluded that this effect was independent of SIRT1 activity and was instead associated with increased hippocampal ATP levels [127].

Table 2 summarizes representative sirtuin modulators according to their molecular target, pharmacological profile, CNS exposure, neurological evidence, and major translational limitations.

## 6. Conclusions

Overall, our bibliometric analysis indicates a marked expansion of research on pharmacological sirtuin modulation in neurological and neuropsychiatric contexts, especially after 2020. The keyword co-occurrence analysis revealed a bibliometric structure organized around several complementary research directions, including epigenetic regulation and protein deacetylation, oxidative stress and inflammation, neurodegeneration and cognitive function, mood regulation, and the rational design of pharmacological modulators.

The central position of terms such as sirtuins, SIRT1, SIRT2, deacetylation, inflammation, oxidative stress, Alzheimer’s disease, molecular docking, and depression suggests a progressive transition from predominantly fundamental molecular research toward more applied neuropsychiatric and pharmacological investigations.

Thus, the bibliometric analysis supports an integrated perspective on sirtuins in neuropsychiatric disorders, extending from molecular and metabolic mechanisms to neurodegeneration, cognitive dysfunction, affective regulation, and therapeutic modulation. These findings also highlight the growing interest in isoform-specific targeting and in the development of more selective sirtuin modulators with potential translational relevance.

Across the reviewed studies, sirtuin-associated compounds were tested under heterogeneous experimental schedules. Some studies used preventive pretreatment designs, particularly in acute toxic or ischemic models, whereas many others evaluated compounds after disease induction or within established genetic, metabolic, or chronic-stress models. This distinction is translationally important because pretreatment studies mainly support prophylactic or resilience-enhancing effects, while post-induction studies provide greater translational relevance than pretreatment designs.

These findings also highlight a central challenge in sirtuin pharmacology: the therapeutic value of sirtuin modulation cannot be reduced to a general strategy of activation or inhibition. Increased SIRT1 or SIRT3 signaling has been associated with neuronal and mitochondrial protection in selected experimental models, including models of Alzheimer-like pathology, Parkinson-like neurodegeneration, cerebral ischemia, and stress-related dysfunction, but these observations should not be interpreted as evidence that activation is uniformly beneficial across diseases or cell types. Likewise, SIRT2 inhibition has reduced proteotoxic and ischemic neuronal injury in some models, whereas SIRT2 activity may restrain inflammatory activation in microglia and contribute to oligodendrocyte and white-matter homeostasis; systemic or prolonged inhibition may therefore produce adverse effects outside the intended neuronal target. Similarly, SIRT6 activation may support neuronal survival and cognitive function in neurodegenerative or hypoperfusion-related models, whereas selective astrocytic SIRT6 inhibition has been associated with antidepressant- and anxiolytic-like effects in stress-related models. Therefore, the same sirtuin isoform may require opposite therapeutic modulation depending on the disease mechanism, cellular target, and pathological stage.

More broadly, the consequences of manipulating a given sirtuin depend on brain region, cell type, disease mechanism and stage, metabolic state, subcellular context, dose, timing, and duration of intervention. Sex-dependent responses have also been reported in selected experimental settings but remain insufficiently characterized. Accordingly, future therapeutic strategies should prioritize disease- and cell-specific target validation rather than assuming a universally beneficial direction of sirtuin modulation.

Dose-dependence represents a major translational limitation in the interpretation of sirtuin modulators. Many compounds discussed in this review are pleiotropic molecules that influence sirtuin signaling indirectly, often through upstream regulation of NAD^+^ metabolism, AMPK, oxidative stress, mitochondrial function, autophagy, or inflammatory pathways. Consequently, the same compound may produce beneficial, neutral, or even harmful effects depending on dose and exposure duration. Resveratrol illustrates this problem clinically: although it is widely described as SIRT1-associated, randomized trials show inconsistent effects on SIRT1 expression, with signals depending on dose, treatment duration, and tissue type. Berberine provides an experimental example of the same issue, because low-dose exposure may engage adaptive antioxidant and survival pathways, whereas higher concentrations or prolonged exposure can increase cytotoxicity and increase dopaminergic degeneration. Therefore, dose should be considered a central variable in sirtuin pharmacology, especially for natural compounds with broad biological activity and limited isoform selectivity.

Neurodegenerative and psychiatric disorders can be conceptualized as dynamic trajectories in which biological systems progressively shift from compensated states toward maladaptive pathological attractors. Sirtuins may influence these trajectories by regulating NAD^+^ metabolism, mitochondrial resilience, oxidative stress, inflammation, proteostasis, autophagy, and synaptic plasticity. Small differences in timing, dose, or cellular context may produce divergent therapeutic outcomes when the biological system is close to a critical transition [135,136]. Accordingly, pharmacological modulation of sirtuins should not be viewed simply as activation or inhibition, but as a context-dependent intervention whose therapeutic effect depends on isoform selectivity, cellular target, disease stage, dose, timing, and duration of exposure.

Overall, the pharmacological evidence for sirtuin modulators in neurological and psychiatric disorders remains predominantly preclinical. With the exception of resveratrol, for which human randomized trials have assessed SIRT1-related outcomes, most compounds have been evaluated in cellular systems, invertebrate models, or rodent models of neurodegeneration, ischemia, neuroinflammation, or stress-related behavior. Therefore, these compounds should be interpreted as experimental modulators rather than clinically validated neuropsychiatric therapeutics.

## 7. Limitations

Several methodological limitations should also be considered when interpreting the available literature. First, publication bias may favor the reporting of positive neuroprotective or behavioral effects, whereas neutral or detrimental findings may be underrepresented. Second, substantial heterogeneity exists across experimental models, including differences in species, cell type, disease induction, treatment timing, dose, route of administration, and outcome measures, which limits direct comparison across studies. Assessment of sirtuin modulation is also inconsistent: some studies quantify sirtuin protein or mRNA expression, whereas others measure substrate acetylation, downstream signaling, or enzymatic activity, while these are not necessarily equivalent indicators of target engagement. In addition, many in vitro studies employ compound concentrations that are substantially higher than those likely to be achieved in vivo or in the human central nervous system, raising concerns regarding supraphysiological exposure and off-target effects.

A major translational gap is the absence of validated, brain-specific biomarkers of sirtuin activity or target engagement in humans, which currently limits interpretation of clinical efficacy and pharmacodynamic response.

Finally, human validation remains limited, with few compounds supported by clinical pharmacokinetic, central target-engagement, biomarker, or efficacy data. Together, these limitations reduce the comparability and translational certainty of the current evidence and emphasize the need for standardized sirtuin activity assays, physiologically relevant exposure ranges, rigorous pharmacokinetic-pharmacodynamic characterization, and replication across complementary disease models, including clinical trials.

## Figures and Tables

**Table 1:** Experimental evidence for SIRT1–SIRT7 in neurological and psychiatric disorders and context-dependent direction of modulation.

Sirtuin	Predominant Subcellular Localization	Experimental Context	Main Experimental Findings	Type of Evidence	Reference
SIRT1	nuclear and cytoplasmic	Neuronal cell cultures; transgenic and other rodent models of Alzheimer-like pathology, synaptic dysfunction, ischemia, neuroinflammation, and chronic stress; human postmortem brain tissue in Alzheimer’s disease and amnestic mild cognitive impairment	Increased SIRT1 signaling improves synaptic plasticity and memory, reduces APP protein processing and amyloid-β accumulation, and enhances neuronal resistance to oxidative, inflammatory, and apoptotic stress. Human postmortem findings also indicate altered SIRT1 regulation in early Alzheimer’s disease, although therapeutic efficacy in humans has not been established.	Cellular Animal Human postmortem tissue	[17,18,19,20]
SIRT2	cytoplasmic	Neuronal cultures, primary mouse cortical neurons subjected to oxygen-glucose deprivation, microglial cells, *Drosophila* Parkinson-like models, rodent Parkinson-like and ischemic models, and APP/PS1 transgenic mice	Inhibition of SIRT2 protects against α-synuclein toxicity in Parkinson-like models and ischemia-associated neuronal death. SIRT2 may suppress inflammatory activation in some microglial contexts.	Cellular Animal	[22,23,24,25,26,27,28]
SIRT3	mitochondrial	Human postmortem Alzheimer’s disease cortex; neuronal cell models and primary hippocampal neurons; genetic and toxin-induced mouse models of Alzheimer-like pathology, Parkinson-like neurodegeneration, cerebral ischemia, and stress-related behavior	Activation or restoration of SIRT3 protects dopaminergic neurons, supports GABAergic inhibitory neurotransmission, improves mitochondrial function, and reduces oxidative injury in models of Alzheimer’s disease, Parkinson’s disease, ischemia, and stress-related behavioral impairment. SIRT3-associated pathways have also been linked to reduced anxiety- and depressive-like behavior in animal models.	Cellular Animal Human postmortem tissue	[30,31,32,33,34,35,36,37,38,39]
SIRT4	mitochondrial	Cellular and rodent models of Alzheimer-like amyloid pathology	Elevated SIRT4 expression promoted neuronal apoptosis, amyloid-β deposition, and memory impairment. SIRT4 reduction attenuated these changes.	Cellular Animal	[40,41,42]
SIRT5	mitochondrial	Amyloid-β-exposed SH-SY5Y human neuroblastoma cells, APP695/PS1-dE9 transgenic mice, microglial models of ischemic inflammation, and mouse models of ischemic stroke and subarachnoid hemorrhage	Increased SIRT5 activity has been associated with mitochondrial resilience, autophagy, reduced oxidative stress, and reduced amyloid-related neuronal injury in some models. Conversely, microglial SIRT5 may promote inflammatory responses after ischemic injury.	Cellular Animal	[43,44,45,46,47]
SIRT6	nuclear	Amyloid-β42-exposed cellular models, brain-specific Sirt6-deficient mice, and astrocyte-specific Sirt6 knockout mice subjected to chronic stress	Activation or preservation of SIRT6 protects neurons from DNA damage, mitochondrial fragmentation, ageing-related dysfunction, and cognitive decline. However, selective SIRT6 deletion in astrocytes produced antidepressant- and anxiolytic-like effects in chronically stressed mice.	Cellular Animal	[48,49,50,51]
SIRT7	nuclear	cellular studies related to genomic stability, ribosomal homeostasis, stress responses, and ageing	Potential neuroprotective relevance has been proposed through maintenance of genomic integrity	Cellular	[52]

APP, amyloid precursor protein; APP/PS1, amyloid precursor protein/presenilin 1; APP695/PS1-dE9, amyloid precursor protein 695/presenilin 1 with exon 9 deletion; Aβ, amyloid beta; Aβ42, amyloid beta 42; DNA, deoxyribonucleic acid; GABA, gamma-aminobutyric acid; SH-SY5Y, human neuroblastoma cell line; SIRT1–SIRT7, sirtuins 1–7.

**Table 2:** Sirtuin modulators with neurological relevance.

Compound	Primary Sirtuin Target	Mechanism/Selectivity	Brain Exposure	Neurological Evidence	Principal Limitations	References
Resveratrol	SIRT1-associated	Pleiotropic modulator; may enhance SIRT1 signaling indirectly through AMPK/NAD^+^-dependent mechanisms	BBB penetration demonstrated in rodents; resveratrol and metabolites detected in human CSF	Broad preclinical evidence; Phase II clinical evidence in Alzheimer’s disease, but efficacy remains unestablished	Poor and variable bioavailability; extensive metabolism	[58,59,128,129]
Eriocitrin	SIRT1-associated indirectly, mainly through eriodictyol; possible SIRT6-associated effects through hesperetin	Indirect, non-selective pleiotropic modulator. SIRT-related effects are demonstrated mainly for metabolites	Not established	Preclinical neurological evidence, largely from eriocitrin-derived metabolites in neuroinflammatory and Alzheimer-like models	No neurological clinical trials	[60,61,62,63,64,65,66,67,68]
Nobiletin	SIRT1-associated	Indirect, non-selective pleiotropic modulator; increases SIRT1/FoxO3a-associated autophagic signaling	Direct brain exposure demonstrated in rodents after oral and intraperitoneal administration	Broad preclinical evidence in cognitive impairment and Alzheimer-like models; limited human cognitive evidence from nobiletin-containing formulations	Indirect modulation; limited target-engagement evidence	[69,70,71]
Ligustilide	SIRT1-associated	Indirect, non-selective pleiotropic modulator; increases SIRT1 expression/signaling and suppresses SIRT1-associated endoplasmic-reticulum stress pathways	Brain (cerebrum and cerebellum) distribution demonstrated in rodents after oral administration	Preclinical evidence in vascular dementia and other neurological injury models	Pleiotropic; causal SIRT1 dependence incompletely established; chemical instability, extensive metabolism	[72,73,74]
Honokiol	SIRT3-associated	Pharmacological SIRT3 activator; increases SIRT3 expression/activity and SIRT3-dependent mitochondrial deacetylation	Crosses the blood–brain barrier and blood–cerebrospinal fluid barrier in rodents	Broad preclinical evidence in Alzheimer-like pathology, cognitive impairment, epilepsy and depression-related models; no established neurological clinical efficacy.	Incomplete sirtuin isoform-selectivity data; extensive metabolism	[75,76,77,78,79,80]
Fisetin	SIRT1-associated	Indirect, non-selective pleiotropic modulator; increases SIRT1/Nrf2/HO-1-associated signaling	Brain penetration demonstrated in mice	Preclinical evidence in brain-ageing, memory-impairment, and depression-like models	Poor oral bioavailability, rapid metabolism, pleiotropic/non-selective pharmacology	[81,82,130]
Piceatannol	SIRT1-associated	Indirect, non-selective pleiotropic modulator; SIRT1/FoxO1-related signaling	Brain exposure in rodents as liposomes	Preclinical evidence in cerebral ischemia/reperfusion and chronic-stress models, with improvements in neurological function, neuronal survival, oxidative stress, anxiety/depression-like behavior, cognition, and neuroinflammation	Pleiotropic/off-target pharmacology	[83,84,85,131]
Berberine	SIRT1-associated	Indirect, non-selective pleiotropic modulator; modulates the AMPK–PGC-1α–SIRT1 axis and mitochondrial/autophagy-related signaling	Brain distribution demonstrated in rodents	Preclinical evidence in Parkinson-like models, with dose- and model-dependent protective versus detrimental effects	Very low oral bioavailability; conflicting preclinical neurotoxicity data	[86,87,88,132,133]
Melatonin	SIRT1/SIRT3-associated	Indirect, non-selective pleiotropic modulator; enhances SIRT1/Nrf2 and Akt–SIRT3–SOD2-associated signaling	Rapid CNS penetration in rodents; oral administration increases melatonin concentrations in human CSF	Broad preclinical evidence in neuroinflammation, depression-like behavior and cerebral ischemia; limited clinical evidence in neurological disorders	Broad pharmacology; sirtuin modulation is only one mechanism	[89,90,91,92,93]
Curcumin	SIRT1-associated	Indirect, non-selective pleiotropic modulator; enhances SIRT1-associated antioxidant, mitochondrial and anti-inflammatory signaling	Detectable in human brain tissue	Extensive preclinical evidence in amyloid toxicity, cerebral ischemia and other neurodegenerative models; limited human evidence for cognitive and mood-related outcomes	Extensive pleiotropy, low bioavailability, indirect mechanism	[94,95,96,97]
Quercetin	SIRT1-associated	Indirect, non-selective pleiotropic modulator; increases SIRT1-associated signaling and modulates ER-stress, antioxidant and anti-inflammatory pathways	Brain accumulation demonstrated in rodents	Preclinical evidence in diabetic encephalopathy and other neurological injury models; human cognitive studies are limited and inconsistent	Indirect and nonselective	[98,99,100]
SRT2104	SIRT1	Direct, relatively selective allosteric SIRT1 activator; binds the SIRT1 N-terminal regulatory region	BBB penetration demonstrated in Huntington’s disease mice	Preclinical neuroprotective efficacy in Huntington-like disease, with additional antidepressant-like and neuroplasticity-related effects in rodent models	Human CNS exposure and neurological efficacy unproven	[104,105,106,107,108,134]
MDL-800	SIRT6	Direct, selective allosteric SIRT6 activator; increases SIRT6 deacetylase activity and promotes deacetylation of H3K9ac/H3K56ac	Incompletely characterized quantitatively	Preclinical evidence in chronic cerebral hypoperfusion/vascular cognitive impairment and demyelination models, with improvements in neuronal survival, cognition and remyelination	Insufficiently characterized	88,89
EX-527	SIRT1	Potent, selective SIRT1 inhibitor; inhibits SIRT1 in the nanomolar range and shows > 200 -fold selectivity over SIRT2/SIRT3. Inhibition is NAD^+^	Direct brain exposure demonstrated in mice	Preclinical efficacy and clinical testing in Huntington’s disease	Reversible liver-enzyme elevations occurred in a Phase II study	[114,115,116,117,118]
AGK2	SIRT2	Relatively selective SIRT2 inhibitor (IC_50_ ~3.5 μM); substantially weaker inhibition of SIRT1/SIRT3	Poor/absent BBB permeability	Preclinical evidence in α-synuclein/Parkinson-like and Huntington models and in cerebral/global ischemic injury	Poor BBB permeability and aqueous solubility	[119,120]
AK-1	SIRT2	Relatively selective, cell-permeable SIRT2 inhibitor; micromolar potency (IC_50_ ~12.5 μM)	Limited	Preclinical evidence in Huntington-like models, tauopathy and in vitro ischemic injury	Modest data	
Compound 33i	SIRT2	Potent and highly selective SIRT2 inhibitor (IC_50_ ~0.57 μM), with markedly weaker activity against SIRT1/SIRT3	Limited	Preclinical effects in depression-like behavior, age-related cognitive decline and APP/PS1 Alzheimer-like pathology	No human data	[26,122,123,124]

AKT, protein kinase B; AMPK, AMP-activated protein kinase; APP/PS1, amyloid precursor protein/presenilin 1; BBB, blood–brain barrier; CNS, central nervous system; CSF, cerebrospinal fluid; ER, endoplasmic reticulum; FoxO1, forkhead box O1; FoxO3a, forkhead box O3a; H3K9ac, histone H3 lysine 9 acetylation; H3K56ac, histone H3 lysine 56 acetylation; HO-1, heme oxygenase-1; IC_50_, half-maximal inhibitory concentration; NAD^+^, nicotinamide adenine dinucleotide; Nrf2, nuclear factor erythroid 2-related factor 2; PGC-1α, peroxisome proliferator-activated receptor gamma coactivator 1-alpha; SIRT1, sirtuin 1; SIRT2, sirtuin 2; SIRT3, sirtuin 3; SIRT6, sirtuin 6; SOD2, superoxide dismutase 2.

## Data Availability

The data supporting the findings of this study are derived from the Web of Science Core Collection. The bibliometric data analyzed in this study are available from the corresponding authors upon request.

## Abbreviations

The following abbreviations are used in this manuscript:

ADP	Adenosine diphosphate
Akt	Protein kinase B
AMP	Adenosine monophosphate
AMPK	AMP-activated protein kinase
APP	Amyloid precursor protein
ATP	Adenosine triphosphate
Aβ	Amyloid beta
BBB	Blood–brain barrier
BDNF	Brain-derived neurotrophic factor
CNS	Central nervous system
CSF	Cerebrospinal fluid
DNA	Deoxyribonucleic acid
ER	Endoplasmic reticulum
ERK	Extracellular signal-regulated kinase
FOXO	Forkhead box O
GABA	Gamma-aminobutyric acid
GPX4	Glutathione peroxidase 4
H_2_O_2_	Hydrogen peroxide
HO-1	Heme oxygenase 1
IC_50_	Half-maximal inhibitory concentration
IL	Interleukin
JNK	c-Jun N-terminal kinase
LPS	Lipopolysaccharide
MAPK	Mitogen-activated protein kinase
MCP-1	Monocyte chemoattractant protein-1
MMP-9	Matrix metalloproteinase-9
MnSOD	Manganese superoxide dismutase
MPTP	1-Methyl-4-phenyl-1,2,3,6-tetrahydropyridine
mTOR	Mechanistic target of rapamycin
NAD^+^	Nicotinamide adenine dinucleotide, oxidized form
NADH	Nicotinamide adenine dinucleotide, reduced form
NAMPT	nicotinamide phosphoribosyltransferase
NF-κB	Nuclear factor kappa B
NLRP3	NOD-like receptor family pyrin domain-containing protein 3
NMN	Nicotinamide mononucleotide
Nrf2	Nuclear factor erythroid 2-related factor 2
PC12	Rat pheochromocytoma cell line PC12
PGC-1α	Peroxisome proliferator-activated receptor gamma coactivator 1-alpha
PI3K	Phosphoinositide 3-kinase
PS1	Presenilin 1
RNA	Ribonucleic acid
ROCK1	Rho-associated coiled-coil-containing protein kinase 1
ROS	Reactive oxygen species
SH-SY5Y	Human neuroblastoma cell line SH-SY5Y
SIRT	Sirtuin
SOD2	Superoxide dismutase 2
TXNIP	Thioredoxin-interacting protein
TGF-β	Transforming growth factor beta
TNF-α	Tumor necrosis factor alpha
6-OHDA	6-Hydroxydopamine
